# Mechanical oscillations orchestrate axial patterning through Wnt activation in *Hydra*

**DOI:** 10.1126/sciadv.abj6897

**Published:** 2021-12-10

**Authors:** Jaroslav Ferenc, Panagiotis Papasaikas, Jacqueline Ferralli, Yukio Nakamura, Sebastien Smallwood, Charisios D. Tsiairis

**Affiliations:** 1 Friedrich Miescher Institute for Biomedical Research, Maulbeerstrasse 66, 4058 Basel, Switzerland.; 2 University of Basel, Petersplatz 1, 4001 Basel, Switzerland.; 3 SIB Swiss Institute of Bioinformatics, 4058 Basel, Switzerland.; 4 Institute of Medical Sciences, University of Aberdeen, AB25 2ZD Aberdeen, UK.

## Abstract

Mechanical input shapes cell fate decisions during development and regeneration in many systems, yet the mechanisms of this cross-talk are often unclear. In regenerating *Hydra* tissue spheroids, periodic osmotically driven inflation and deflation cycles generate mechanical stimuli in the form of tissue stretching. Here, we demonstrate that tissue stretching during inflation is important for the appearance of the head organizer—a group of cells that secrete the Wnt3 ligand. Exploiting time series RNA expression profiles, we identify the up-regulation of Wnt signaling as a key readout of the mechanical input. In this system, the levels of Wnt3 expression correspond to the levels of stretching, and Wnt3 overexpression alone enables successful regeneration in the absence of mechanical stimulation. Our findings enable the incorporation of mechanical signals in the framework of *Hydra* patterning and highlight the broad significance of mechanochemical feedback loops for patterning epithelial lumens.

## Teaser

Mechanochemical feedback drives patterning in *Hydra* tissue during regeneration.

## INTRODUCTION

Animal bodies and organs display an overwhelming variability of forms, yet their development relies on a relatively small repertoire of key processes such as folding, branching, and lumenization (*1*, *2*). These morphogenetic processes often generate mechanical forces that can in turn guide cellular behaviors, thus creating mechanochemical feedback loops (*3*). In such cross-talk, the patterning phenomena bridge organization scales as tissue properties like elasticity or curvature influence individual cell biochemistry and vice versa. Epithelial lumen expansion is a characteristic case, where the fluid accumulation in the cavity increases pressure globally and affects patterning of the surrounding epithelium. Differential fate decisions of individual cells are then often driven by local heterogeneities of the global tissue properties and can further modify them (*4*). Mechanically driven inflation and deflation events have been observed to influence the patterning of mammalian blastocysts (*5*, *6*), lung alveoli (*7*), otic vesicles (*8*), and diverse organoid systems (*9*, *10*). To understand the conserved general principles of this luminal patterning, anatomically simple systems with well-defined cell differentiation processes, and amenable to experimental manipulation, are particularly handy. The critical questions are what type of mechanical signals the cells react to, and how they modify their identity, i.e., gene expression profile. Regenerating *Hydra* tissue spheroids are such a simple, experimentally tractable system, derived from an animal whose phylogenetic position makes it suited to uncover broadly conserved mechanisms (*11*). Here, mechanics and cell fate decisions coincide, but their connection has remained elusive thus far (*12*, *13*).

**Fig. 1.**
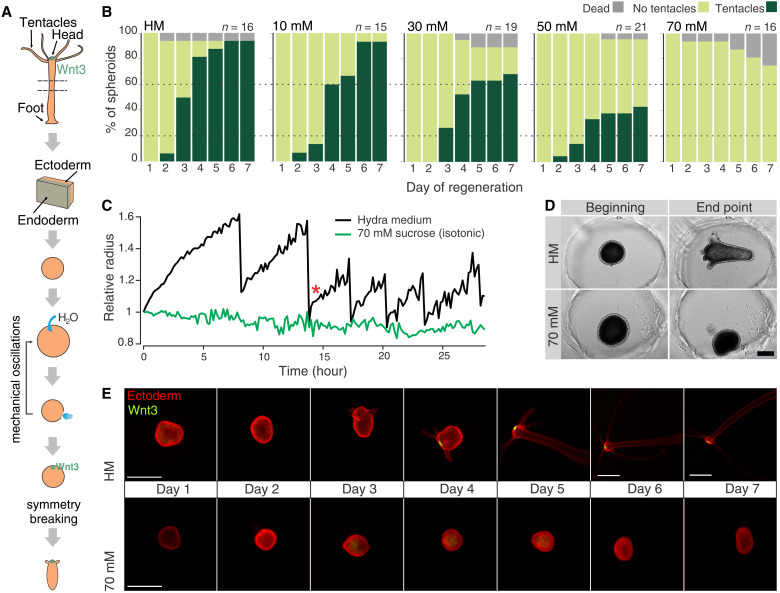
The impact of mechanical stimulation on head regeneration. (**A**) Spheroid preparation and regeneration. (**B**) Regeneration success decreases in media with increasing osmolarities. Cumulative plots for *n* animals from three independent experiments. The concentration of sucrose in *Hydra* medium (HM) is indicated above each plot, 70 mM being isotonic. (**C**) Quantifications of the size of representative spheroids from movies S1 and S2. Note the absence of oscillations in isotonic conditions. Asterisk marks phase I/II transition. (**D**) Snapshots of the initial (*t* = 0 hours) and final time points (*t* > 50 hours) from movies S1 and S2, demonstrating the inability to regenerate in isotonic conditions (70 mM sucrose). (**E**) Fluorescent imaging of representative spheroids from a Wnt3-reporter line in normal and isotonic conditions without oscillations. There is a known delay in the reporter visibility, which causes it to be observable only after tentacle appearance (*22*). Scale bars, 500 μm.

*Hydra* is a simple organism organized along a single oral/aboral axis. Two epithelial layers, the ectoderm and the endoderm (also termed epidermis and gastrodermis), form a tubular structure with a mouth surrounded by tentacles on one end and a foot on the opposite. Wnt signaling is critical for the patterning of the animal with ligands expressed on the oral end, the hypostome area (*14*). Ectopic activation of Wnt signaling induces ectopic axes formation (*15*). Moreover, Wnt signaling is critical during the morphallactic regeneration of *Hydra* (*16*). When a small fragment is taken from the body of *Hydra*, the two-layered epithelial tissue folds into a spheroid with a lumen in the center. This spheroid gradually re-establishes the full body plan as the head (hypostome) with tentacles, and the foot (peduncle and basal disc) of the animal appear, marking the opposite ends of its body axis (Fig. 1A). The uniformity of the epithelial cells is first broken when a small subpopulation stably expresses the Wnt3 ligand, differentiating into the head organizer which guides the morphogenesis of the surrounding tissue (*17*, *18*).

Previous work has shown cycles of osmotically driven mechanical oscillations accompanying the regeneration process of such small fragments (*19*) (movie S1). Since *Hydra* is a freshwater animal, the cells need to maintain their osmotic balance against water that enters from the hypotonic environment. They excrete the surplus water into the spheroid lumen (*20*). Thus, the spheroid inflates, and the tissue is stretched at the same time (fig. S1, A to D). Once the stretching reaches a critical threshold, the tissue ruptures causing the spheroid to deflate. Variable numbers of inflation and deflation cycles have been observed before a change of this oscillatory behavior marks the break of symmetry and mouth establishment (*21*). For example, experimental evidence shows that it is possible to bias the position of the future head but only before the oscillatory behavior transition (*22*). It has also been demonstrated that the mouth opening is stabilized after this transition (*23*), resulting in earlier release of the accumulated liquid and smaller spheroid expansions. The oscillation pattern thus changes from a high-amplitude, low-frequency regime (termed phase I) to a low-amplitude and high-frequency one (phase II; Fig. 1C). However, whether and how the mechanical oscillations are linked to the establishment of the hypostome organizer remained unknown.

In the current work, we used imaging and mechanical perturbations to investigate the role of inflation and deflation cycles and found that the stretching level of the tissue during inflation is essential for the downstream patterning of the epithelia. Then, through a time series RNA sequencing (RNA-seq) of the regenerating spheroids, we identify the impact of mechanical signals specifically for the appearance of the oral end of the body axis, with the *Wnt3* gene being a key target. The expression of *Wnt3* is quantitatively related to the amount of tissue stretching, and the expression of this gene enables patterning in the absence of mechanical stimulation. These results facilitate incorporation of mechanical signals in a coherent framework that will explain *Hydra* patterning. Moreover, it underscores the evolutionary conserved connection between Wnt signaling and mechanical stimuli and indicates that a lumen expansion could be a general mechanism for epithelial patterning.

## RESULTS

### Mechanical oscillations are required for the head organizer establishment

Given the osmotic nature of inflation mechanism, oscillations can be perturbed by manipulating environmental osmotic pressure. Including additional osmolytes (e.g., sucrose) in the medium slows down the spheroid inflation rate in a concentration-dependent manner (*19*), and, in isotonic conditions, the oscillations cease completely (fig. S1, E to G, and movie S2). Using this approach, we observed a progressive loss of regeneration capacity (measured by the appearance of new tentacles) with increasing osmolarity. Under isotonic conditions (70 mM sucrose), spheroids completely fail to regenerate (Fig. 1, B to D). However, they remain viable for several days and can reinitiate the regeneration program when transferred back to normal medium (fig. S1, H and I), indicating that isotonic environment is not inherently toxic. Rather, it suggests that the absence of mechanical input puts a temporary halt to the regeneration program.

To answer whether mechanical input is required for the emergence of the Wnt3^+^ organizer cells, we used a reporter line, where green fluorescent protein (GFP) is expressed under the Wnt3 promoter recapitulating the normal Wnt3 expression pattern (*24*). All spheroids lacked GFP^+^ foci under isotonic conditions (Fig. 1E and fig. S1J). Thus, mechanical input is required for the differentiation of the head organizer rather than the execution of the downstream morphogenetic program of tentacle development. Unexpectedly, even in the absence of oscillations, many spheroids elongate in the isotonic medium over time (fig. S1K). Acquiring an asymmetric shape is, therefore, not a hallmark of molecular symmetry breaking, as previously suggested (*21*). Head regeneration in bisected animals is also hindered in isotonic conditions, indicating that some level of mechanical stimulation is a general requirement for *Hydra* regeneration (fig. S1, L and M, and movie S3).

### The readout of mechanical oscillations is continuous rather than periodic

**Fig. 2.**
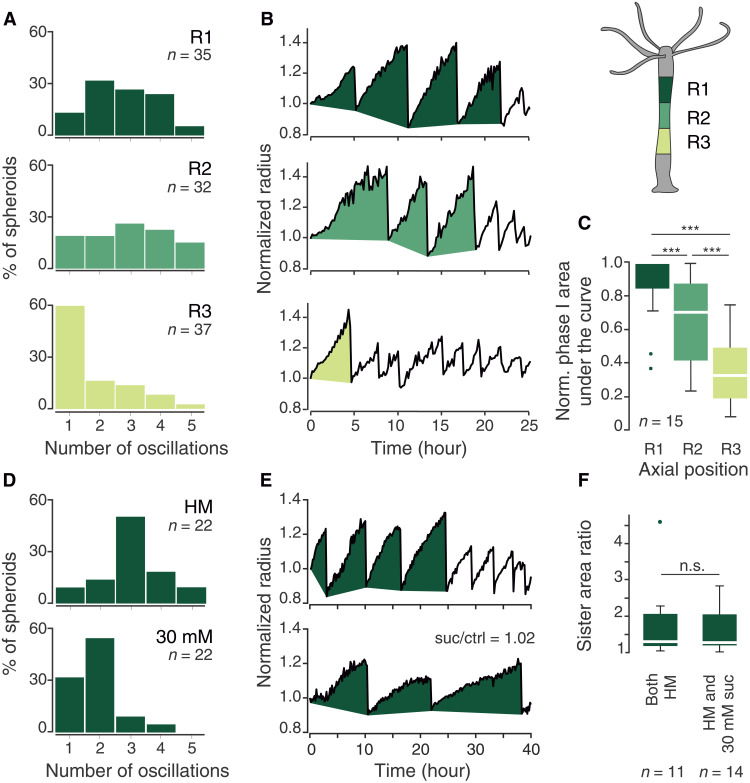
Features of mechanical oscillations important for regeneration. (**A**) Distributions of the number of phase I oscillations for spheroids from different axial positions (R1 to R3, see schematic). Data of *n* samples pooled from three independent replicates. (**B**) Representative examples of oscillation patterns for three spheroids derived from different axial positions of the same individual. Filled parts correspond to phase I oscillations. (**C**) Quantification of the phase I area under the curve for 15 animals from (A) for which data were available for all three positions. Measurements for each animal were normalized to the R1 position. (**D**) Comparison of the oscillation number distribution for R1 spheroids in HM versus 30 mM sucrose (*n* samples from five independent experiments). (**E**) Representative example of two spheroids from the identical axial position of a single animal (sister spheroids) showing a similar phase I area in different media. (**F**) Area ratios for pairs of sister spheroids. They were either both cultured in HM or one of the pair was put into 30 mM sucrose. The ratios for each pair are always normalized to the sister with a smaller phase I area, irrespective of the conditions (*n* samples pooled from three independent experiments). Data in (C) and (F) were analyzed using the Mood’s median test. ***P < 0.0005 (p_R1/R2_ = 1.0322 × 10^−4^, p_R1/R3_ = 2.9061 × 10^−08^, p_R2/R3_ = 8.7409 × 10^−04^).

To understand the role of these mechanical oscillations, we examined the range of phase I cycle numbers among spheroids. Such differences can result from intrinsic symmetry breaking variability among the tissue pieces. To investigate the source of this variability, we looked at the behavior of spheroids originating from different axial positions. Classical grafting experiments have demonstrated gradients of head formation and inhibition capacity along the axis. Tissue originating closer to the head is able to establish a new organizer more efficiently when grafted, but, as a host environment, it is less conducive to a new organizer appearance (*25*, *26*). Therefore, we examined whether the axial origin of the regenerating spheroids also affects their mechanical dynamics. Spheroids derived from tissue closer to the head require more cycles before breaking the symmetry than those originating further away. These results thus likely reflect the persistence of an inhibitory property bestowed by the proximity of an existing head (Fig. 2, A and B). In contrast, other oscillation properties, such as cycle duration or inflation rate, do not vary significantly among the measured axial positions (fig. S2, A to D).

Having established that mechanical input is indispensable for successful regeneration and that the number of phase I oscillations correlates with an inhibitory axial gradient, we then asked whether spheroids require a specific number of cycles to be able to regenerate (a “periodic counting” model). Alternatively, they might sense the overall amount tissue stretching experienced during the oscillations, disregarding deflation events (a “continuous” model). To distinguish between these options, we imaged spheroids in a medium with intermediate osmolyte concentration (30 mM sucrose), which slows down the rate of inflation and, consequently, prolongs each cycle’s duration (fig. S2, E to H). Only spheroids from the R1 position were used since they have the highest number of cycles on average. Assuming a counting mechanism, all cycles should be executed irrespective of their duration. Instead, we observed a decrease in the average number of phase I oscillations in higher osmolarity (Fig. 2D). This argues that a continuous mechanism relying on the overall amount of tissue stretching is more plausible. This metric can be approximated by quantifying the area under the plot of phase I oscillations (hereafter phase I area; Fig. 2, B and C). Sister spheroids, derived from the same axial position and from the same animal, tend to have a very similar phase I area even in different medium osmolarities, where the number of oscillations is different (Fig. 2, E and F). These results underscore the crucial role of sustained mechanical tissue stretching during inflation for successful symmetry breaking and regeneration.

### Head, but not foot, regeneration transcriptional program shows a strong mechanical dependence

**Fig. 3.**
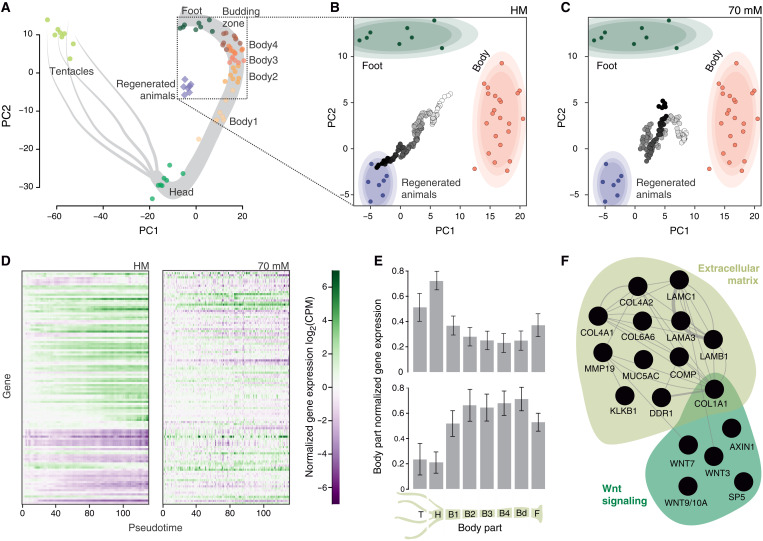
Transcriptional changes in regenerating spheroids. (**A**) Principal components analysis (PCA) plot of different body parts based on their transcriptional identity. Full animals regenerated from spheroids (harvested 72 hours after cutting) are also included. Dotted box indicates the area enlarged in the following panels. (**B** and **C**) Projections of the spheroid time course data into the body PCA space with (B) and without mechanical input (C). Shaded ovals around the body parts (body2 to body4) and regenerated animals data correspond to 90, 80, and 70% Gaussian confidence intervals (light to dark shading). The color of the projected time course data represents the pseudotime progression (from white to black). Each individual dot corresponds to a single spheroid. (**D**) Heatmaps of the behavior of the top 10% (*n* = 113) genes affected by lack of mechanical simulation in control and isotonic conditions. For each gene, the initial (*t*^0^) log_2_(CPM, counts per million) value was subtracted. (**E**) Average expression patterns in the body for the increasing (top) and decreasing genes (bottom) from (D). The expression data for each gene were normalized to the body part with the highest expression of that gene, before averaging per body part. Error bars show 95% confidence intervals. (**F**) Subnetworks of genes showing functional enrichment within the up-regulated genes, affected by lack of oscillations. Edges indicate physical, genetic, and predicted interactions or coexpression in human cells (data from genemania.org).

To understand the molecular impact of tissue stretching, we performed a time course of RNA-seq in spheroids (fig. S3, A to D) under both control and isotonic conditions. This identified ~2300 genes exhibiting at least a twofold change in expression over the course of normal regeneration. Strikingly, we could not detect any periodic changes associated with spheroid deflations (fig. S3B), further corroborating the previous findings of a continuous readout. Neither was there an indication of the samples experiencing osmotic stress in 70 mM sucrose (fig. S3C). We then combined these temporal data with a positional RNA-seq of isolated body pieces from individual animals (fig. S4, A and B). The body parts’ transcriptomes allowed us to map gene expression profiles onto positional identities along the axis (Fig. 3A). When projected into this map, the time course data of >150 individual spheroids per condition revealed their regeneration trajectories. Under normal conditions, spheroids follow a relatively straightforward path from a body-like state toward a regenerated animal with both ends of the axis established (Fig. 3B). However, in isotonic medium, without mechanical input, the trajectory lacks directionality and follows an undulating path that eventually turns toward a foot-like identity (Fig. 3C).

We then looked at the top 10% of genes (*n* = 115) most severely affected by the removal of mechanical input (fig. S3E) to gain insight into the transcriptional changes behind the observed developmental differences. Most (*n* = 73) of these genes were increasing over time during normal regeneration, while a smaller portion (*n* = 37) showed a decreasing trend (Fig. 3D). These groups of genes also have very different axial expression profiles. The rising genes show a clear enrichment in the head region, consistent with their role in setting up the body part most affected by the lack of mechanical oscillations. On the other hand, the group of decreasing genes shows enrichment in the undifferentiated body column, highlighting the failure to differentiate properly when oscillations are inhibited (Fig. 3E). Looking at functional annotations within the rising cluster (tables S1 and S2), we found an enrichment of extracellular matrix related genes, which likely reflects the tissue remodeling that has to occur to tolerate the mechanical stress and to regenerate. Several components of the Wnt signaling pathway were also enriched, including three ligands of the canonical Wnt signaling (Fig. 3F).

**Fig. 4.**
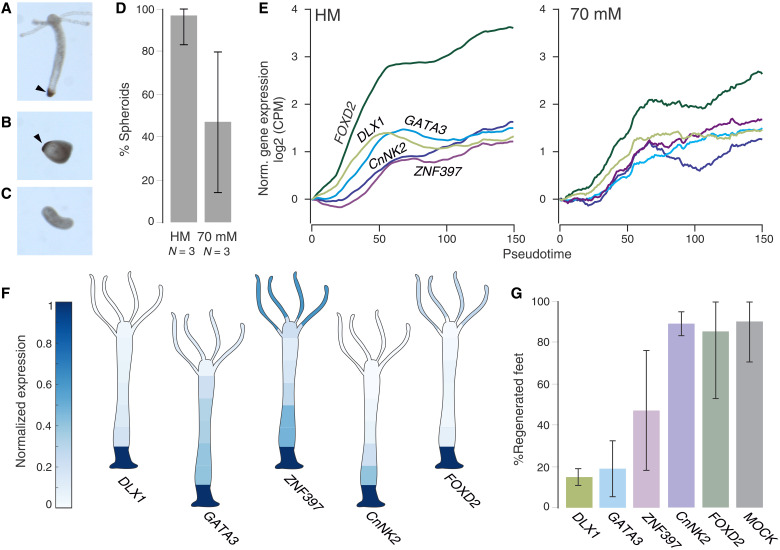
Foot regeneration without mechanical stimulation. (**A**) Representative spheroid that regenerated under control conditions shows peroxidase staining in the basal disc (foot). (**B**) Similar foot peroxidase staining was observed in some spheroids cultured in 70 mM sucrose in the absence of a head. (**C**) Elongated spheroids developing in the absence of mechanical stimulation do not always have a peroxidase marked foot. (**D**) Quantification of foot marker presence in control (HM) or isotonic medium (70 mM sucrose) grown *Hydra* spheroids. Plot shows the average percentages of peroxidase-positive spheroids from three independent experiments. Error bars indicate 95% confidence intervals. Forty-two spheroids were examined in total for the control and 26 for isotonic conditions. (**E**) Expression patterns of the small cluster of foot-specific transcription factors in control and isotonic medium (70 mM sucrose). Lines indicate moving averages (moving window width is 36 pseudotime points). (**F**) Average body expression patterns of these genes in homeostatic animals (averaged from eight animals and normalized for each gene to the body part with highest expression level). (**G**) Results of foot regeneration in bisected animals upon knockdown of these transcription factors. Averages from three independent experiments are shown.

Before further examining the head regeneration requirements, we focused on the aboral axis end. The trajectory of spheroids in the isotonic medium moving toward a foot-like identity suggests an interesting possibility to regenerate a foot without the head. This would mean that the axis ends can be largely independently established and differ in the needs for mechanical stimulation. When examined for the characteristic foot peroxidase staining, about 50% of these spheroids are positive for the marker (Fig. 4, A to D). Moreover, looking at the expression pattern of known early foot markers, such as *CnNK2* (*27*) and *Dlx1* (*28*), we found a small cluster of foot-specific transcription factors with a coordinated temporal behavior (Fig. 4, E and F). All these genes show a steep early rise in transcript abundance, followed by a plateau at high expression levels. This progression is not abolished by the absence of mechanical stimulation, although it becomes noisier (Fig. 4E), which might account for the decrease in the success of foot regeneration under isotonic conditions. Knockdown, when successful (fig. S4C), of several of these factors (*Dlx1* and *Gata3*) also demonstrates their importance for the foot establishment, as it decreases the ability to regenerate a foot after bisection (Fig. 4G). Our data thus support the view that the two poles of the oral/aboral axis function as separate (yet connected) organizing centers (*29*) with differential requirements for mechanical stimulation when being established de novo in the context of tissue spheroids.

### Wnt3 expression functions as a quantitative readout of tissue stretching

**Fig. 5.**
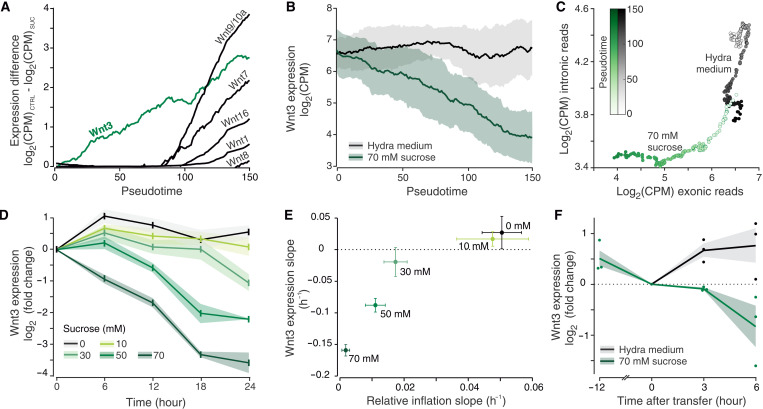
The relationship between tissue stretching and Wnt3 expression. (**A**) Temporal development of the expression differences of canonical Wnt ligands between control and isotonic conditions. (**B**) Wnt3 expression patterns in HM and isotonic medium. In both (A) and (B), the lines indicate moving averages (moving window width is 36 pseudotime points). Shaded area represents a moving SD (window width identical to the mean). (**C**) Scatter plot of intronic versus exonic reads for the Wnt3 transcript from the single-sphere RNA-seq time course. Data were smoothened using a moving average with a window size of 36 pseudotime points. (**D**) Wnt3 expression dynamics in different osmolarities measured with quantitative polymerase chain reaction. Data for each condition were normalized to the initial time point. Shown are averages from three independent biological replicates. Shaded areas represent SEM. (**E**) Relationship between the spheroid inflation rate (slope) and the slope of Wnt3 expression (for raw data, see figs. S1A and S5C). The error bars indicate 95% confidence intervals. (**F**) Wnt3 expression in spheroids, preincubated in isotonic medium (70 mM sucrose), upon transfer to HM or continued incubation. Only expanded spheroids before deflation were collected from HM at 3 and 6 hours after transfer. Data from three independent biological replicates normalized to the transfer time point. Lines indicate averages, shaded area SEM.

Coming back to the impact of tissue stretching on Wnt signaling, we noticed that the lack of mechanical stimulation affected the expression dynamics of all the canonical Wnt signaling ligands (Fig. 5, A and B, and fig. S5A). These genes are activated sequentially during normal regeneration in bisected animals (*16*, *28*), and we observed a similar progression for spheroids. After the pseudotime point 80, most of the transcripts rapidly increase, reflecting a successful establishment of the mouth organizer. However, none of these changes take place without mechanical stimulation. *Wnt3* expression has a behavior distinct from the other Wnt ligands (Fig. 5B). Previous studies have shown that *Wnt3* is up-regulated very early in response to injury (*30*, *31*) and then sustained throughout the regeneration process. Yet, we observe that, in isotonic conditions, its expression fails to be sustained and rapidly decreases. While such dynamics can be explained by tissue stretching acting to either stabilize the transcript or activate *Wnt3* transcription, the analysis of intronic versus exonic reads favors the latter option (Fig. 5C and fig. S5C). Although both conditions show comparable levels of exonic reads at the beginning of the time course, transcription is clearly more active in *Hydra* medium (HM), as indicated by the levels of intronic reads. As time elapses, the transcript dynamics of the control sample are still predominantly transcription-driven up to the pseudotime point ~100, whereafter mRNA stability/degradation has a more prominent role. This is probably a result of switching on more complex regulations while the organizer is established. Without tissue stretching, however, the intronic counts quickly decrease and plateau at baseline levels, suggesting that the gene transcription is switched off. Subsequently, the exonic reads continue decreasing, as, in the absence of transcription, the temporal development is solely determined by the mRNA degradation. We therefore hypothesized that the *Wnt3* gene transcriptional output functions as a readout of the mechanical input. To test this hypothesis, we performed a quantitative polymerase chain reaction (qPCR) time course under different osmolarities, corresponding to different intensities of mechanical stimulation. As expected, the ability to sustain *Wnt3* expression was anticorrelated with the mechanical input strength (Fig. 5, D and E, and fig. S5, D and E). To further verify that *Wnt3* transcription is coupled to tissue stretching, rather than tearing upon deflation, we measured *Wnt3* mRNA levels in spheroids that were reintroduced to HM after 12-hour preincubation in an isotonic medium (70 mM sucrose). This initial period without oscillations served to lower the *Wnt3* transcript levels. Our understanding predicts that, when reintroduced to HM, *Wnt3* levels would begin to rise because of tissue stretching before the first spheroid collapse. This is what we observed (Fig. 5F), further arguing for the *Wnt3* transcription to be connected with tissue stretching, rather than deflation, in the context of spheroid oscillations. Since head-regenerating halves of bisected animals also show a decrease of regeneration success in an isotonic medium without oscillations, we investigated whether this results in lower *Wnt3* mRNA levels. Whole-mount in situ hybridization of such samples has confirmed this assumption. We observe that, in most bisected animals without mechanical stimulation, the initial expression fails to be sustained (fig. S5F).

### Wnt3 expression is sufficient to rescue the absence of mechanical stimulation

**Fig. 6.**
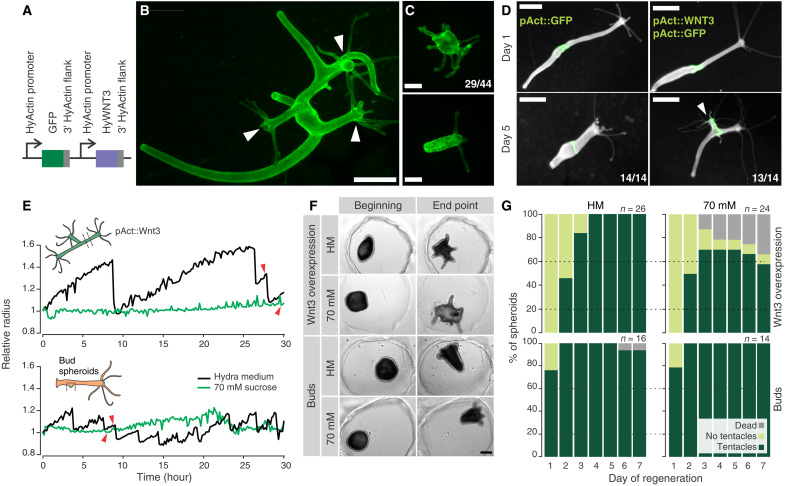
Rescue of head regeneration in the absence of mechanical stimulation. (**A**) Schematic of the construct used to generate transgenic animals. Both *GFP* and *WNT3* expression is driven by *Hydra* actin promoter and flanked by a 3′ untranslated region of the *Hydra* actin gene. GFP serves as a transgenesis marker. (**B**) Example of a fully transgenic individual expressing the construct in the ectoderm and showing a typical branched morphology with ectopic heads (arrowheads). (**C**) Examples of regenerated Wnt3-overexpressing spheroids. Numbers indicate the incidence of the multitentacle phenotype in regenerating Wnt3-overexpressing spheroids. Data were pooled from three independent experiments. (**D**) Ectopic head (arrowhead) induction upon grafting tissue (green) from Wnt3-overexpressing animal (right) versus assimilation of tissue from a control GFP-expressing animal (left). Numbers indicate the number of grafts with identical phenotype to the one shown for each condition (data pooled from three independent experiments). Fluorescence images merged with dark-field images of the entire animals. (**E**) Quantifications of the spheroid behavior for movies S4 to S7 representative of regeneration rescue in the presence of Wnt3. Red arrowheads indicate the emergence of the first tentacle. (**F**) Snapshots of the initial time points (*t* = 0 hours) and regenerated animals (*t* > 50 hours) from movies S4 to S7. (**G**) Spheroids derived from Wnt3-overexpressing animals and buds regenerate equally well irrespective of the presence or absence of mechanical stimulation. Cumulative plots of *n* samples combined from three independent replicates. Scale bars, (B and D) 1 mm and (C and F) 200 μm.

We then asked whether providing Wnt3 would be sufficient to rescue regeneration in the absence of mechanical stimulation. To this end, we generated animals overexpressing *Wnt3* in the whole ectoderm (Fig. 6, A to D), which showed a branched morphology with multiple heads (Fig. 6B). This phenotype is similar to the one observed after β-catenin overexpression (*32*). Spheroids derived from these animals were able to regenerate (Fig. 6C), sometimes producing regenerates with multiple tentacles that would later resolve into several heads. Moreover, body column pieces of these animals showed organizer-like properties when transplanted into a wild-type (wt) context, as they induced ectopic head formation (Fig. 6D).

These animals enable us to examine the sufficiency of *Wnt3* expression for proper regeneration in the absence of mechanical oscillations. Spheroids derived from the *Wnt3*-overexpressing animals were also able to regenerate in isotonic conditions, showing that artificially sustaining the *Wnt3* expression circumvents the need for mechanical stimulation. While the oscillatory behavior is not necessary for these spheroids to regenerate, we also noticed that they are able to inflate more before collapsing than the wt sphreoids (Fig. 6E and fig. S5G). One possible explanation of these observations would be a change of tissue mechanical properties upon Wnt3 overexpression. Successful development without oscillations was also observed in an orthogonal experiment with early buds. In this approach, spheroids are derived from early buds and already have an organizer that renders them unaffected by the lack of mechanical stretching (Fig. 6, E to G, and movies S4 to S7). We therefore conclude that sustaining *Wnt3* expression is both necessary and sufficient for successful *Hydra* head organizer regeneration and that it is made possible by its stretching-dependent transcriptional activation.

## DISCUSSION

Despite the fact that mechanical oscillations are a prominent feature of *Hydra* regeneration from small tissue pieces, their role in this process had long remained elusive. We show that the mechanical stretching, caused by these inflation/deflation cycles, is the critical cue for the de novo differentiation of an organizer in this context. Similarly, even when animal halves are establishing a new hypostome after bisection, some level of inflation-driven stretching is required. In this case, the success rate decreases markedly when the animals are placed in isotonic medium that prevents the osmotic inflations. While we cannot completely exclude the possible side effects of our experimental intervention beyond changing the oscillatory behavior, we do not see evidence for the outcome of the experiments being stress-driven or dependent on the osmolyte used. Since mechanical oscillations also occur in the reaggregates of cells from dissociated *Hydra* (*21*), the emergence of organizers there is likely accomplished through a similar mechanism. Moreover, pulsating behavior has also been observed during *Hydra* embryogenesis. After the two epithelial layers are formed, the embryos display rhythmic expansions and contractions that endure until hatching (*33*). Thus, various modes of the oral organizer emergence in *Hydra* are associated with mechanical stimulation in the form of tissue stretching.

**Fig. 7.**
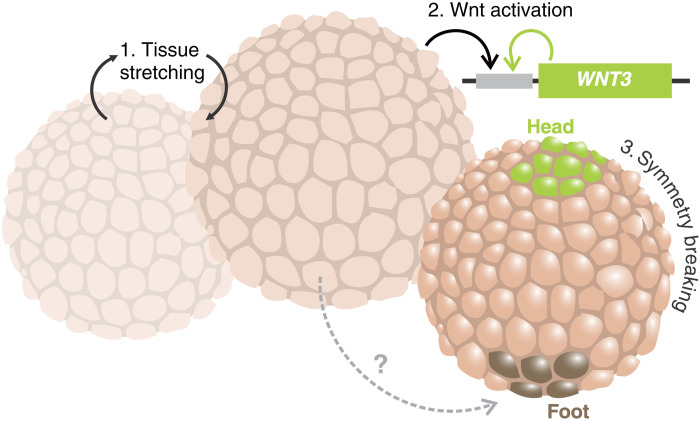
The role of mechanical oscillations in *Hydra* whole-body regeneration. Mechanical inflation/deflation cycles have a critical role in providing tissue stretching, which activates Wnt3 transcription during *Hydra* regeneration. The expression of this gene can later be self-sustained as is pivotal for specifying the oral pole of the body axis.

The key molecular pathway, which specifies the *Hydra* mouth organizer, is the canonical Wnt signaling (*14*). It has been shown that its ectopic activation can trigger supernumerary organizers in the body column and consecutively, ectopic axis formation (*15*). Among all its ligands, Wnt3 has the most prominent role, as it is the first to be expressed when a new organizer emerges (*16*), and its expression can be maintained through a positive-feedback loop (*24*). We see that mechanical oscillations are critical for regulating the *Wnt3* gene expression (Fig. 7), with the levels of transcripts depending quantitatively on mechanical stimulus. Up-regulation of Wnt signaling output after mechanical stimulation has been observed during the gastrulation of *Nematostella* (*34*) and zebrafish embryos (*35*) as well. In these cases, the link of the Wnt cascade with mechanics appears to be the phosphorylation of β-catenin. In *Hydra*, such a link is still unknown, but the identification of *Wnt3* as the key target gene enables further investigation of the mechanism that transforms a physical signal into biochemical output. Previous work on the Wnt3 promoter has already identified several regulatory elements (*22*, *30*) that can serve as a departure point for a promising investigation. Nevertheless, the fact that the hypostome axial organizer emergence is linked molecularly with tissue stretching prompts us to reevaluate the prevailing model on *Hydra* axial patterning.

*Hydra* axial patterning can be largely explained by a reaction diffusion model proposed originally by Gierer and Meinhardt (*36*). An activator/inhibitor pair, with the diffusion coefficient of inhibitor being higher than the activator one, is anticipated to be at the core of a mechanism that guarantees local self-activation and long-range inhibition of the organizer identity. In essence, the mechanistic explanation is similar to the one proposed by Turing (*37*), but despite its success in explaining a variety of phenomena and despite the identification of Wnt3 as the likely head activator (*38*), no activator/inhibitor pair suitable for the particular model has been found. The recent identification of the transcription factor Sp5 as a possible repressor (*30*) also highlighted the need to expand our thinking on how the short-range activation and long-range inhibition can be materialized. Several theoretical models, both in other systems (*39*) and *Hydra* (*40*), have also already considered the option that these signals might be of mechanical nature. Since our results demonstrate a concrete connection between a mechanical property and the expression of a crucial morphogen, they will be valuable for future modeling efforts trying to bridge the current biochemical framework with tissue mechanics.

The integration of mechanics with biochemical patterning cues remains an open question and is closely related with the spatial specification of the organizer. While all cells of the spheroid are being stretched, only some of them will finally differentiate into the head organizer. We hypothesize that this choice can be driven by the heterogeneity of cells response to mechanical stretching in the tissue, which could result in different levels of *Wnt3* expression across the spheroid. This model is consistent with recent experimental work indicating a role for supracellular actin fibers in positioning the organizer (*41*, *42*). Moreover, the foot (aboral) pole of *Hydra* body axis emerges in our results as a largely autonomous counterpart to the mouth (oral) pole. Not only can it be established without mechanical oscillations but also seems to be specified faster than the mouth organizer. It is therefore possible that the initially foot-primed cells create an exclusion zone for the hypostome differentiation. Such mechanism would ensure that even if the opposite poles of the axis can emerge independently, they would be properly positioned relative to each other.

Since fluid-filled lumens are one of the leitmotifs of animal morphogenesis (*43*), our system offers an excellent platform to study the general rules of lumen formation and function, particularly the relationship between pressure-dependent cell stretching and cell fate decisions (*3*, *4*). Several developmental contexts where lumen expansion affects cell fate changes have been identified. For example, proper alveolar differentiation in the embryonic lung depends on sufficient mechanical stimulation from the pressure of amniotic fluid (*7*). However, whether the downstream signaling pathways (in this case, fibroblast growth factor) also provide a quantitative readout of mechanical stimulation similar to what we describe is not known. While such mechanisms could work with many signaling molecules, the case of Wnt is particularly intriguing thanks to its ancient evolutionary past. The origin of Wnt signaling coincides with the origin of multicellularity (*44*), thus suggesting that its mechanosensitivity in *Hydra* and other cnidarians (*34*) reflects an early evolutionary connection. Whereas first multicellular animals likely had a poor signaling repertoire, the combination of biochemical signals with mechanical cues may have enabled body plan elaboration (*45*), as we report here for *Hydra* spheroids.

Moreover, mechanical input driving Wnt expression is a widespread phenomenon in diverse developmental contexts (*46*–*48*), as well as in disease (*49*). In particular, tissue stretching seems to activate Wnt-signaling ligands transcriptionally in some of these cases. We thus suggest that this might be a conserved mode of Wnt signaling activation through mechanical stimulation. As we learn more about the upstream mechanosensitive pathways in *Hydra*, it will be interesting to see whether only the regulatory logic is conserved in higher metazoans or also the molecular players and how these evolutionary choices could have affected the emergence of more complex body plans. Together, the mechanism transforming mechanical stimuli into a quantitative transcriptional output, which we have identified in *Hydra*, represents a potentially conserved implementation of mechanochemical cross-talk in basic morphogenetic processes such as luminogenesis and tissue stretching.

## MATERIALS AND METHODS

### Animal strains and culture conditions

All experiments were performed using either the AEP or 105 strains of *Hydra vulgaris*. Animals were kept in HM [1 mM CaCl_2_, 0.2 mM NaHCO_3_, 0.02 mM KCl, 0.02 mM MgCl_2_, and 0.2 mM tris-HCl (pH 7.4)] at 18°C and fed with freshly hatched *Artemia* nauplii three times a week. Individuals without buds, starved for 24 hours, were used for experiments, if not indicated otherwise. All experiments were performed in accordance with ethical standards and the Swiss national regulations for the use of animals in research.

### Spheroid preparation

Details of spheroid generation, imaging, and oscillation analysis can be found in a previously published protocol (*50*). Animals were bisected (exact position of the initial cut for different experimental designs is detailed below) and one or several thin tissue rings were obtained by sequential cutting. The rings were then split in two to three rectangular pieces that were left to close for ca. 2 to 2.5 hours in HM or dissociation medium [DM; 3.6 mM KCl, 6 mM CaCl_2_, 1.2 mM MgSO_4_, 6 mM sodium citrate, 6 mM sodium pyruvate, 4 mM glucose, and 12.5 mM *N*-tris(hydroxymethyl)methyl-2-aminoethanesulfonic acid (pH 6.9)] at room temperature (RT). Properly closed spheroids (typical diameter, 300 to 500 μm) were then separated from the pool and used for further experiments.

### Live imaging of developing spheroids

Multichamber LabTek slides (Nunc) were used for imaging. Spheroids were placed in individual agarose wells (ca. 1 mm in diameter) within the chambers to retain them in the field of view. The wells were created by coating the bottom of the chambers with a 2- to 3-mm-thick layer of 1% agarose in HM and puncturing holes using a 1-ml micropipette tip after the gel solidified. The chamber was then filled with the desired imaging medium. In case of sucrose treatments, the agarose gel contained sucrose in concentrations equal to the overlaying medium. For inhibitor treatments, drug concentration in the imaging medium was adjusted, taking the volume of agarose into account. To prevent diluting the imaging medium in the chamber, closed spheroids were first transferred to a dish with bigger volume (ca. 10 ml) of the imaging medium and positioned in the imaging wells only after this wash. Samples were imaged every 10 min at 21°C under bright-field illumination using the Nikon Ti2-Eclipse microscope with a 10× CFI Plan Apochromat Lambda objective (Nikon) and iXon Ultra 888 electron-multiplying charge-coupled device (EMCCD) camera (Andor). Images were acquired in the 1024 × 1024 format, with pixel size of 1.3 μm.

### Quantification of oscillation parameters

Bright-field images were batch segmented in Fiji (*51*). A course median filter (radius = 60 pixel) was first applied, followed by Phansalkar local thresholding (radius = 50 pixel, *r* and *k* parameters = 0) and another round of median filtering (radius = 20 pixel). The area of segmented objects bigger than 5000 μm^2^ including holes was then measured. Radius was calculated from these values, assuming a spherical object. Radius data for each sample were normalized to the starting value and further processed in MATLAB using custom functions to extract the slope, amplitude, and period of the phase I oscillations. The number of oscillations and phase I/II transition was determined manually by two independent experimenters using a criterion of at least 30% decrease in the amplitude and doubling of the oscillation frequency.

### Quantifying average ectoderm thickness

Spheroids from the line *AEP ecto pAct::eGFP* (*52*) were prepared and imaged as previously described. Samples were imaged every 10 min at 21°C using the Nikon Ti2-Eclipse microscope with 488-nm laser illumination (Toptica iBeam smart, 500 mW) with a 20× CFI Plan Apochromat Lambda objective (Nikon) and iXon-Ultra-888 EMCCD camera (Andor). Images were acquired in the 1024 × 1024 format with pixel size of 0.65 μm. Care was taken to always image the equatorial plane of the spheroid. Images were then processed in Fiji. First, the contrast was adjusted and the images were then smoothed by two iterations of the 3 × 3 unweighted smoothing function and segmented using the global segmentation function. For each image, two segmentations were generated—one of the entire area of the optical section, including the tissue, and another one of the inner cavity. The radius was then calculated as √(area/π). For each image, the average tissue thickness was taken to be the difference of the two radii.

### Plasmid electroporation for sparse GFP labeling of cells

Plasmid DNA of the HotG plasmid (*52*) was extracted from liquid bacterial culture using the EndoFree Plasmid Maxi Kit (QIAGEN). Twenty to 25 animals per treatment were transferred into an electroporation cuvette (Gene Pulser/Micro Pulser Electroporation Cuvette 0.4-cm gap or Bio-Rad electroporation cuvettes 4-mm gap, VWR), washed with Milli-Q water twice and then incubated at 4° for 30 min followed by RT for 30 min. Residual water was removed, and 200 μl of a plasmid solution (10 μg of plasmid and 10 mM Hepes to 200 μl) was added to each cuvette. Animals were incubated with this solution for 5 min. After relaxation of the animals, two pulses (150-V range) were applied for 75 ms (Gene Pulser II with RF module, Bio-Rad). After electroporation, 500 μl of ice-cold HM was added to the animals. Animals were carefully transferred into a new petri dish filled with prechilled HM. Electroporations were performed in this manner on 3 days (0, 2, and 4). Animals were used for experiments when first GFP^+^ cells appeared (~ 1 week after the last electroporation).

### Scoring regeneration and Wnt center appearance in spheroids

Spheroids from the lines *AEP ecto pAct::eGFP* (*52*) and *AEP ecto pAct::dsRed, pWnt3::eGFP* (*24*) were prepared as described above. Animals were bisected at 20% of the body length below the head, and one or two rings per animal were generated. Spheroids were left to close in HM. Similar to previous studies (*19*), we used sucrose to alter the medium osmolarity. Sucrose was dissolved in HM to the desired concentration [0, 10, 20, 30, 50, and 70 mM (isotonic)], supplemented with antibiotics [kanamycin (50 μg/ml) and streptomycin (100 μg/ml)] and filter sterilized. Spheroids were randomly split among the conditions (0 and 70 mM only for Wnt3-reporter line) in 24-well plates, kept at 18°C, and scored for the presence of tentacles in 24-hour intervals for 7 days. In addition, the presence of the organizer was assessed in the Wnt3-reporter line spheroids by fluorescence imaging. The Zeiss AxioZoom V.16 fluorescent stereomicroscope with the Zeiss Axiocam MRm CCD camera was used. Last, the regenerated animals/surviving spheroids were fixed on the seventh day of the time course and stained to determine the presence of the foot.

### Experiments with restarted oscillations

*AEP ecto pAct::eGFP* spheroids were prepared as described above, and the whole population of properly closed spheroids was transferred to 70 mM sucrose in HM to prevent mechanical oscillations. Samples were kept in this medium for 72 hours at 18°C. Surviving spheroids were then randomly split into two groups and transferred either to 70 mM sucrose again (controls) or to HM, allowing the mechanical oscillations to restart. Both populations were monitored daily for tentacle appearance as in the previous experiments.

### Scoring head regeneration in bisected animals

*AEP ecto pAct::eGFP* animals were bisected at 50% body length, and the halves were kept separately in the wells of 24-well plates at 18°C. Tentacle appearance was monitored in 24-hour intervals in the foot halves for 5 days.

### Peroxidase foot staining

The staining was performed as previously described (*53*). Briefly, animals were relaxed in 2% urethane in HM and fixed with 4% paraformaldehyde (PFA) in HM at 4°C overnight. Animals were then washed in phosphate-buffered saline + 0.1% Tween 20 (PBST) for 5 min and stained for 15 min in the staining solution (0.02% diaminobenzidine, 0.03% hydrogen peroxide, and 0.25% Triton X-100 in PBS). To stop the enzymatic reaction, samples were washed once again in PBST for 15 min. The whole staining procedure was performed at RT with mild agitation.

### Imaging and quantification of oscillations in spheroids of different axial origin

To obtain spheroids from different axial positions, the head of the *AEP ecto pAct::GFP* animals was cut away at ~10% of the body length, and three rings of tissue were obtained sequentially. Each ring was split into two pieces. Pieces were incubated at RT for 2.5 hours in DM while closing. To allow backtracking of the piece identity, sister pieces from each ring were kept in separate wells of a 24-well plate, noting the axial position and animal of origin. Similar arrangement was followed for imaging, which otherwise proceeded as described above. Quantifications of oscillation parameters from obtained time-lapse images were also performed as outlined previously.

### RNA-seq time course of developing spheroids

Over the course of 10 hours, few dozens of spheroids were prepared every hour from the 105 strain, let to close for 2 hours in DM, and then transferred to either HM or HM with 70 mM sucrose. Eight spheroids from each batch were then collected 3 and 13 hours after cutting the last batch, thus creating a time course spanning a window of 22 hours. Spheroids were collected individually in 96-well plates in 45 μl of RLT+ buffer (QIAGEN) and stored at −80°C for a later RNA purification. This was performed using the Zymo Direct-zol MagBead reagents following a modified manufacturer protocol. Briefly, 45 μl of Zymo Binding Buffer and 4 μl of MagBeads was added to the samples/RLT+ buffer and incubated for 10 min. Beads were washed three times with 100% ethanol and incubated with 12.5 μl of deoxyribonuclease I (Zymo) for 10 min. The RNA was captured back on the beads using 100 μl of MagBead PreWash buffer (10 min incubation). After washing the beads three times with 100% ethanol, elution was performed with water (13 μl) at 55°C for 15 min. All above incubations were performed at RT on a plate shaker unless specified. The resulting RNA quality was assessed using a bioanalyzer or TapeStation RNA HS kit and concentration determined using Qbit. cDNA amplification was performed using the SmartSeq2 approach as per the original protocol (*54*). Full-length cDNA was processed for Illumina sequencing using Tagmentation with an in-house purified Tn5 transposase (*55*): 1 ng of amplified cDNA was tagmented in TAPS-DMF buffer [10 mM TAPS (pH8.5), 5 mM MgCl2, and 10% DMF], at 55°C for 7 min. Tn5 was then stripped using SDS (0.04% final concentration), and tagmented DNA was amplified using Phusion High-Fidelity DNA Polymerase (Thermo Fisher Scientific). PCR was performed in the Phusion HF buffer, with a first extension at 72°C for 3 min, followed by 10 cycles of amplification (95°C, 30 s; 55°C, 30 s; and 72°C, 30 s). Commercial Nextera XT indexes were used for the PCR amplification (1:5 dilution). Final libraries were sequenced on a HiSeq2500 (50 cycles single-end) and demultiplexing performed using a standard Illumina bcl2fastq2 pipeline.

### Positional RNA-seq

Animals of the 105 strain were first bisected at the midpoint between the head and the budding zone. The halves created this way were bisected again in the middle between the head and the previous cut, or the previous cut and the budding zone, respectively, thus creating “body2” and “body3” segments. Head with tentacles and budding zone with the foot were then removed from the remaining pieces to generate “body1” and “body4” segments. Last, the budding zone was separated from the foot using the change of endoderm coloration as a guideline for sectioning. Tentacles were also separated from the head, trying to remove as much of the tentacle tissue as possible. The individual segments were lysed immediately after being cut in 350 μl of RL buffer (Single Cell RNA Purification Kit, Norgen), supplemented with 1% β-mercaptoethanol, frozen on dry ice, and stored at −80°C for later RNA isolation. Tentacles of a single animal were pooled as one sample. RNA extraction was performed according to the manufacturer’s instructions. Downstream processing of the isolated RNA was identical with the previously described experiment using the SmartSeq2 protocol and in-house Tn5 transposase. RNA from animals regenerated from spheroids was also isolated and sequences as described here. The spheroids were prepared from the 105 strain, as described in the time course sequencing experiment above and left to regenerate in HM for 72 hours. Individual whole regenerated animals were then collected in 350 μl of RL buffer in three independent replicates (two to three animals per replicate).

### RNA-seq data alignment and preprocessing

The SmartSeq2 libraries for the spheroid RNAseq generated a total of ~8.9 billion 50–nucleotide (nt) long reads (accounting for an average sequencing depth of ~25 million reads per spheroid). The Illumina Smartseq2 libraries for the positional and regenerated animals RNA-seq generated a total of 1.7 billion and 250 million 50-nt long reads, respectively, corresponding to an average depth of ~27 million reads per segment replicate and ~ 31 million reads per regenerated animal. After demultiplexing, reads were aligned against the *Hydra* genome guided by transcriptome annotation [National Center for Biotechnology Information (NCBI) *Hydra* vulgaris assembly *Hydra*_RP_1.0, NCBI *Hydra* vulgaris annotation release 102] using STAR (*7*) version 2.5.0 with command line parameters: *--outSJfilterReads Unique --outFilterType BySJout --outFilterMultimapNmax 5 --alignSJoverhangMin 8 --alignSJDBoverhangMin 4 --outFilterMismatchNoverLmax 0.1 --alignIntronMin 20 --alignIntronMax 1000000 --outFilterIntronMotifs RemoveNoncanonicalUnannotated --seedSearchStartLmax 50 --twopassMode Basic --genomeChrBinNbits 12 --genomeSAsparseD 2 --quantMode GeneCounts*. Samples with library sizes smaller than 5 million reads were discarded from all subsequent analyses. In total, 32 of 352 spheroid libraries and 1 of 72 animal segment libraries were removed at this step. The produced gene count tables for all remaining samples were library normalized after excluding from the size factor calculation the top 5 percentiles of highly expressed genes. As the samples along the 22-hour time course of the spheroids were obtained in two collection sessions (one session for time points 1 to 11 hours and one session for time points 12 to 22 hours), a batch effect was introduced. This manifested as a slight discontinuity both in principal components analysis (PCA) projections of the samples and in select time course expression profiles of individual genes. To mitigate this effect, we took advantage of the fact that spheroids develop asynchronously (median pseudotime spread per collection point of 1.6 hours; see the “Pseudotime ordering of spheroids” section) allowing us to pinpoint samples across batches that nearly overlap in terms of dynamics. The correlation (Spearman’s rho) of samples from adjacent time points across the two collection series (16 samples collected at 10 to 11 hours and 14 samples collected at 12 to 13 hours) was calculated to identify pairs of top 3 mutual nearest neighbors (MNNs), corresponding to inferred overlapping samples across batches. These MNN pairs were used to calculate average gene-specific shifts that were then applied as correction factors on all samples from the two batches. This simple strategy efficiently removed the observed discontinuities both in sample projections and individual gene profiles. The corrected spheroid expression values are used for all downstream analyses.

### Pseudotime ordering of spheroids

Individual spheroids evolve asynchronously, with samples collected at a specific experimental time exhibiting not only technical but also internal developmental time variation. To recover the underlying gene expression dynamics, it is therefore necessary to order the samples along an axis corresponding to the temporal evolution of the system and to obtain latent coordinates for each sample on that axis. Our strategy for pseudotime ordering relied on first identifying data features that are smooth functionals of the time variable and subsequently using those features to obtain a one-dimensional (1D) projection of the spheroids on a basis that corresponds to the pseudotime axis. This procedure was applied separately to the regular medium and isotonic 22-hour spheroid time course datasets sampled at 1-hour intervals. We begin by selecting the top 50% most variable genes, according to a within-dataset mean-variance trend fit. Each of the first 50 principal components (*P*) of this filtered dataset was fit against a generalized additive model (GAM) using time (*t*) as the independent variable [function *gam* from the *mgcv* CRAN library, call: *gam(P ~ s(t), method = “GCV.Cp”, gamma = 1.0)*]. The dataset is then reconstructed using only components with gam-fit false discovery rate (FDR)–adjusted *P* < 0.05 as the rest will contain information almost orthogonal to time dynamics. Next, we iterated over all 16, overlapping, 7-hour time windows and repeated the gam-fitting procedure for the expression of all genes **(G)** to identify individual features that are informative for time ordering within the corresponding timeframe [function call: *gam(G ~ s(t, k = 4), method = “GCV.Cp”, gamma = 1.25)*]. For each 7-hour time window, 100 1D isomap embeddings were performed using random subsets of half of the respective selected gene expression profiles. Within each 7-hour window, pseudotime was estimated as the mean of the returned 1D coordinates from the isomap iterations scaled to 7 hours plus a shift corresponding to the first window time point. This procedure returns between 1 and 7 pseudotime estimates per spheroid (depending on how many 7-hour windows cover the corresponding collection time). A final consensus pseudotime was obtained using the weighted average of the individual estimates with weights determined by the number and GAM-fit significance of the genes selected in each respective 7-hour window.

### Projection of segment, regenerated animal, and spheroid data on a common subspace

For the common subspace projection of spheroid, segment and regenerated animal data expression values were first log_2_-transformed after smoothing using a pseudocount of 8 to shrink effect sizes of lowly expressed genes. The pseudotime-ordered spheroid data were further smoothed using a moving average with a window size of 16 (corresponding to a real-time window of ~2 hours) since spheroids exhibit considerable technical variation. We then obtained a single eigenvector basis by applying PCA on the animal segment data (base R function *prcomp* with parameters *center = TRUE, scale = FALSE*). Only the intersection of the genes with at least a twofold change in gene expression (max absolute delta of 1 for the log-transformed values) in both the segment and spheroid datasets was used. Last, the spheroid and regenerated animal data were projected back to the eigenvector space determined by the animal segment data.

### Differential analysis of gene expression in spheroids in hypotonic and isotonic media conditions

To identify genes that are differentially expressed in the hypotonic (HM) and isotonic (70 mM sucrose medium) conditions, we fitted a GAM on the delta of the two pseudotime-ordered expression time series. First, to allow comparisons between the two conditions, we had to account for the fact that, after filtering low-depth libraries, we end up with a larger number of spheroids in HM conditions compared to the 70 mM conditions (170 versus 150 spheres; see the “RNA-seq data alignment and preprocessing” section). We downsampled the HM data by interpolating to 150 sampling points (*t*) to acquire two equal-length time series. The difference Δ of the two signals for every gene was scaled and fitted to a GAM [function *gam* from the *mgcv* CRAN library, call: *gam(*Δ ~ *s (t, k = 8), method = “GCV.Cp”, gamma = 1.0)*]. In addition, we calculated effect sizes for each gene between the two conditions as the sum of the absolute values of the difference between the two mean-normalized signals, excluding the highly variable first three time points. This procedure allows us to identify genes that differ either in terms of scale or in terms of shape in their gene expression dynamics. Both the FDR-adjusted *P* values from the fit (<1 × 10^−6^) and the calculated effect sizes (>40) were used to select for genes differentially expressed in the two conditions.

### Analysis of the changing genes sensitive to mechanical stimulation

The 2269 genes that change their expression at least twofold during normal regeneration were clustered in five clusters using the *kmeans* function in MATLAB. Before clustering, the gene expression time series of each gene was mean-normalized. To select the top genes sensitive to the removal of mechanical stimulation, we only considered the ones with adjusted *P* < 10^−6^. From this pool, the top 10% were selected on the basis of the sum of differences (*n* = 113). To enable the functional analysis of these genes, their mammalian homologs were then annotated using the available data from *Hydra* genomics databases (see table S1). Gene Ontology term enrichment analysis was then performed using GeneMania (genemania.org) separately for members of clusters 3 and 5. Since there were only three genes that were members of other clusters, these were left out of the analysis.

### Knockdowns of foot-specific transcription factors

Gene knockdowns were performed according to a protocol based on (*56*). Briefly, 20 to 30 animals per RNA interference (RNAi) treatment were incubated at 4°C for 1 hour and then transferred into an electroporation cuvette (Gene Pulser/Micro Pulser Electroporation Cuvette 0.4-cm gap, Bio-Rad or electroporation cuvettes 4-mm gap, VWR) and washed twice with chilled Milli-Q water. Residual water was removed, and 200 μl of the small interfering RNA (siRNA) (siRNAs were ordered as 20–base pair RNA duplexes from Integrated DNA Technologies) solution was added to each cuvette. Each siRNA was diluted in ddH_2_O to a final concentration of 1.35 μM per treatment. Animals were incubated with the siRNA solution for 5 min. After relaxation of the animals, two pulses (150-V range) were applied for 50 ms (Gene Pulser II with RF module, Bio-Rad). After electroporation, 500 μl of ice-cold recovery medium [80% HM, 20% (v/v) DM] was added to the animals. Animals were carefully transferred into a new Petri dish filled with prechilled recovery medium. The next day, the animals were transferred into fresh HM. Three electroporations were performed in this manner with 1 day for recovery in between successive rounds. The animals were then bisected at 50% body length 2 days after the last electroporation and stained for peroxidase 3 days after bisection, as described above. Total numbers of animals used in all three replicates were 27 *Dlx1*, 29 *Gata3,* 30 *Znf397*, 30 *CnNK2*, 33 *FoxD2*, and 33 mock.

### Real-time PCR verification of RNAi efficiency

Knockdowns of foot-specific transcription factors and bisections were performed as described above. On the third day after the bisection, three foot-regenerating halves were collected per sample and lysed in 350 μl of RL buffer with 1% β-mercaptoethanol (Single Cell RNA Purification Kit, Norgen), frozen on dry ice, and stored at −80°C. After collecting all the samples, RNA was isolated as outlined in the instructions for the kit. The concentration and purity of the extracted RNA was verified using NanoDrop 1000 (Thermo Fisher Scientific). Next, cDNA was prepared with the Oligo(dT)12-18 Primer (Thermo Fisher Scientific) using the High-Capacity cDNA Reverse Transcription Kit (Applied Biosystems) as per manufacturer’s instructions. The qPCR reactions were carried out using the StepOnePlus Real-Time PCR cycler (Thermo Fisher Scientific) with a standard run method. Each reaction contained 10 ng of cDNA, 1× the Platinum SYBR Green qPCR SuperMix-UDG w/ROX (Invitrogen) master mix with 0.25 μM primers in a total volume of 25 μl. The sequences of the primer pairs used are given in Table 1 below. The quantification of gene expression was performed using the StepOne software v 2.3 (Thermo Fisher Scientific).

**Table 1.** Sequences of primers used in the experiment.

**Gene**	**Forward primer (5′ → 3′)**	**Reverse primer (5′ → 3′)**
*GAPDH*	GACTTGGCCGTATTAACTTGAGC	CTACAAACAAGACGCCCTATTCG
*DLX1*	GTGAAGATGACGATGAAGATTTAAC	AACGATTTAATTCTCGGAGCTG
*GATA3*	TAAACCAAAGAGGAGATTGTCAC	ATACTGGTTCACCACTTCCA
*ZNF397*	AACCAACTCATTCTAGCTGC	TGTGCTTCAATCATTCGTGT
*NK2*	GGTTTAAGTTGCATGGTTGC	AACTTGGAGATTCACACTTAGG
*FOXD2*	GCAGTTTTAACGAAGAACACC	TGTCGGGTGAAGCTAAAATG

### Real-time PCR time course of Wnt3 expression in different osmolarities

Spheroids of the 105 strain were prepared as described previously, randomly split into five groups, and incubated in media with different sucrose content (0, 10, 30, 50, and 70 mM) at RT. Every 6 hours, a sample of 10 spheroids was randomly taken from each of the conditions, lysed in 350 μl of RL buffer with 1% β-mercaptoethanol (Single Cell RNA Purification Kit, Norgen), frozen on dry ice, and stored at −80°C. After collecting all the samples, RNA was isolated as outlined in the instructions for the kit. The concentration and purity of the extracted RNA was verified using NanoDrop 1000 (Thermo Fisher Scientific). Next, cDNA was prepared with the Oligo(dT)12-18 Primer (Thermo Fisher Scientific) using the High-Capacity cDNA Reverse Transcription Kit (Applied Biosystems) as per manufacturer’s instructions. The qPCR reactions were carried out using the StepOnePlus Real-Time PCR cycler (Thermo Fisher Scientific) with a standard run method. Each reaction contained 10 ng of cDNA, 1× the Platinum SYBR Green qPCR SuperMix-UDG w/ROX (Invitrogen) master mix with 0.25 μM primers in a total volume of 25 μl. The quantification of gene expression was performed using the StepOne software v 2.3 (Thermo Fisher Scientific). Table 2 below shows the sequences of primers used.

**Table 2.** Sequences of the primers used.

**Gene**	**Forward primer (5′ → 3′)**	**Reverse primer (5′ → 3′)**
*GAPDH*	GACTTGGCCGTATTAACTTGAGC	CTACAAACAAGACGCCCTATTCG
*WNT3*	TGCAGAAGGAATACGACTGGG	TGCTGGCTGTTGTAATAATTGGG

### Real-time PCR time course of Wnt3 expression upon transfer in different media

Spheroids of the 105 strains were prepared as describe above and incubated for 12 hours in isotonic medium (HM with 70 mM sucrose). After this initial incubation, the surviving spheroids were randomly split into two groups, one of which was transferred to HM, while the other one was kept in the isotonic medium. Random samples of nine spheroids were taken from the populations in the beginning of the experiment, after the initial incubation and 3 and 6 hours after transfer. In case of the HM population, care was taken to only include expanded spheroids. Samples were lysed in 350 μl of RL buffer with the addition of 1% β-mercaptoethanol (Single Cell RNA Purification Kit, Norgen), frozen on dry ice, and stored at −80°C. Further processing of the samples was identical to the previous experiment.

### Generation of transgenic animals overexpressing Wnt3

The transgenesis of animals was performed as previously described (*24*, *52*). Briefly, two-cell stage embryos were injected with the pAct::GFP, pAct::Wnt3 construct and left to develop. After hatching, the polyps were reared separately and screened for the expression of the transgenesis marker GFP. Fully transgenic animals were then obtained from buds originating in the transgenic cell patches.

### Rescue experiments with buds and Wnt-overexpressing spheroids

Animals of the line *AEP ecto pAct::eGFP* with buds in stages 3 and 4 [staging according to (*57*)] were selected, and tissue rings containing the forming buds were cut. Only the budding fragment of the ring was then excised and allowed to form a spheroid in HM. Given the branched multiheaded morphology of the Wnt-overexpressing *AEP ecto pAct::eGFP, pAct::Wnt3* line, it was not possible to cut tissue rings in a specific axial position. Rings were harvested from sufficiently long body fragments instead and cut into two to three fragments as usual. Fragments were left to close in HM. Both types of spheroids were then split between 0 and 70 mM sucrose in 24-well plates and scored for tentacle appearance, as detailed above.

### Whole-mount in situ hybridization

In situ hybridization was performed according to previously published protocols (*58*) with minor modifications. Briefly, animals were fixed at 4°C in 4% PFA overnight and then dehydrated in a series of 5 min washes in 25-50-75-100% methanol and incubated overnight in methanol at −20°C. Rehydration then followed by successive 10 min washes of 75-50-25-0% methanol in PBST. Samples were then treated for 10 min with a proteinase K solution (10 μg/ml), and the reaction was stopped by incubation with glycine (4 mg/ml) for 10 min. After 2× 5-min washes with PBST and 2× 5-min washes with 0.1 M triethanolamine solution, samples were treated with acetic anhydride for 2× 5 min, washed in PBST (2× 5 min), and refixed in 4% PFA for 20 min. Thorough washing with PBST (5× 5 min) then followed, and the animals were heat treated for 30 min at 80°C. Following preincubation with the hybridization solution for 2 hours at 55°C, digoxigenin (DIG)–labeled RNA probe dissolved in hybridization solution was added. We used the same *Wnt3* probes previously described (*14*). The samples were incubated with the probes for 3 days and then washed with a series of 75-50-25-0% solutions of hybridization buffer in 2× saline sodium citrate buffer (SSC). After blocking the samples in 20% sheep serum for 2 hours at 4°C, an incubation with the alkaline phosphatase–conjugated anti-DIG antibody followed (overnight, 4°C). To remove the unbound antibody, 8× 1-hour washes with maleic acid buffer were performed, followed by an overnight wash in the same buffer. The next day, samples were treated by NTMT and levamisole, as detailed in the original protocol. Last, the chromogenic reaction was performed using the bromochloroindolyl phosphate–nitro blue tetrazolium Color Development Substrate (Promega) according to the manufacturer’s instructions.

### Transplantation experiments

Transplantation were performed on glass needles, hand-pulled from glass capillaries. Recipient wt AEP animals were bisected at 50% body length, and the foot half was driven on the needle longitudinally. For controls, a ring of tissue from the same axial position was cut from the *AEP ecto pAct::eGFP* animals and threaded on the needle. Since it is not possible to match axial positions in the Wnt3-overexpressing animals, we used rings of tissue coming from anywhere in the branched body columns of these animals. The head half of the recipient was then added, thus sandwiching the transplant between the host body halves. Pieces were then secured with a piece of parafilm to prevent sliding of the needle and left for ~2 hours in HM to establish adhesion. After this time, healed grafts were carefully slid off the needle using fine forceps and left to recover. The success of transplantation was evaluated after 24 hours. Successful transplant was then assessed for ectopic head formation on day 5 after transplantation.

### Statistical analysis

All experiments have been performed in at least three independent replicates. Details about sample numbers are given in the figures or descriptions of the relevant experiments. Since the nature of the collected data often led to distributions that were not Gaussian, we used the nonparametric Mood’s median and Wilcoxon rank sum tests. These tests were performed as two-sided in all cases. For pairwise comparisons, presented in fig. S1, we used a one-sided paired Student’s *t* test. Other relevant details are given in the figure legends and experiment descriptions. In all box plots, the central mark corresponds to the median, and the bottom and top box edges indicate the 25th and 75th percentiles, respectively. Whiskers show the range of the data points not considered outliers. Outliers are defined as points that have a value of less than *q*_1_ – *w* × (*q*_3_ – *q*_1_) or more than *q*_3_ + *w* × (*q*_3_ – *q*_1_). Here, *w* is the maximum length of the whisker, and *q*_1_ and *q*_3_ correspond to the 25th and 75th percentiles, respectively.

## Notes

View/request a protocol for this paper from *Bio-protocol*
