# Supplementary material for: Mechanical oscillations orchestrate axial patterning through Wnt activation in Hydra
Source: Sci Adv. 2021 Dec 10;7(50):eabj6897. doi: 10.1126/sciadv.abj6897 (PMC8664257; doi:10.1126/sciadv.abj6897)
Supplement: Supplementary file 1 — Figs. S1 to S5 Tables S1 to S3 Legends for movies S1 to S7 [file sciadv.abj6897_sm.pdf]

Science Advances
AAAS

Supplementary Materials for

Mechanical oscillations orchestrate axial patterning through Wnt activation in *Hydra*

Jaroslav Ferenc, Panagiotis Papasaikas, Jacqueline Ferralli, Yukio Nakamura, Sebastien Smallwood, Charisios D. Tsiairis*

*Corresponding author. Email: charisios.tsiairis@fmi.ch

Published 10 December 2021, *Sci. Adv.* **7**, eabj6897 (2021)
DOI: 10.1126/sciadv.abj6897

The PDF file includes:

Figs. S1 to S5
Tables S1 to S3
Legends for movies S1 to S7

Other Supplementary Material for this manuscript includes the following:

Movies S1 to S7

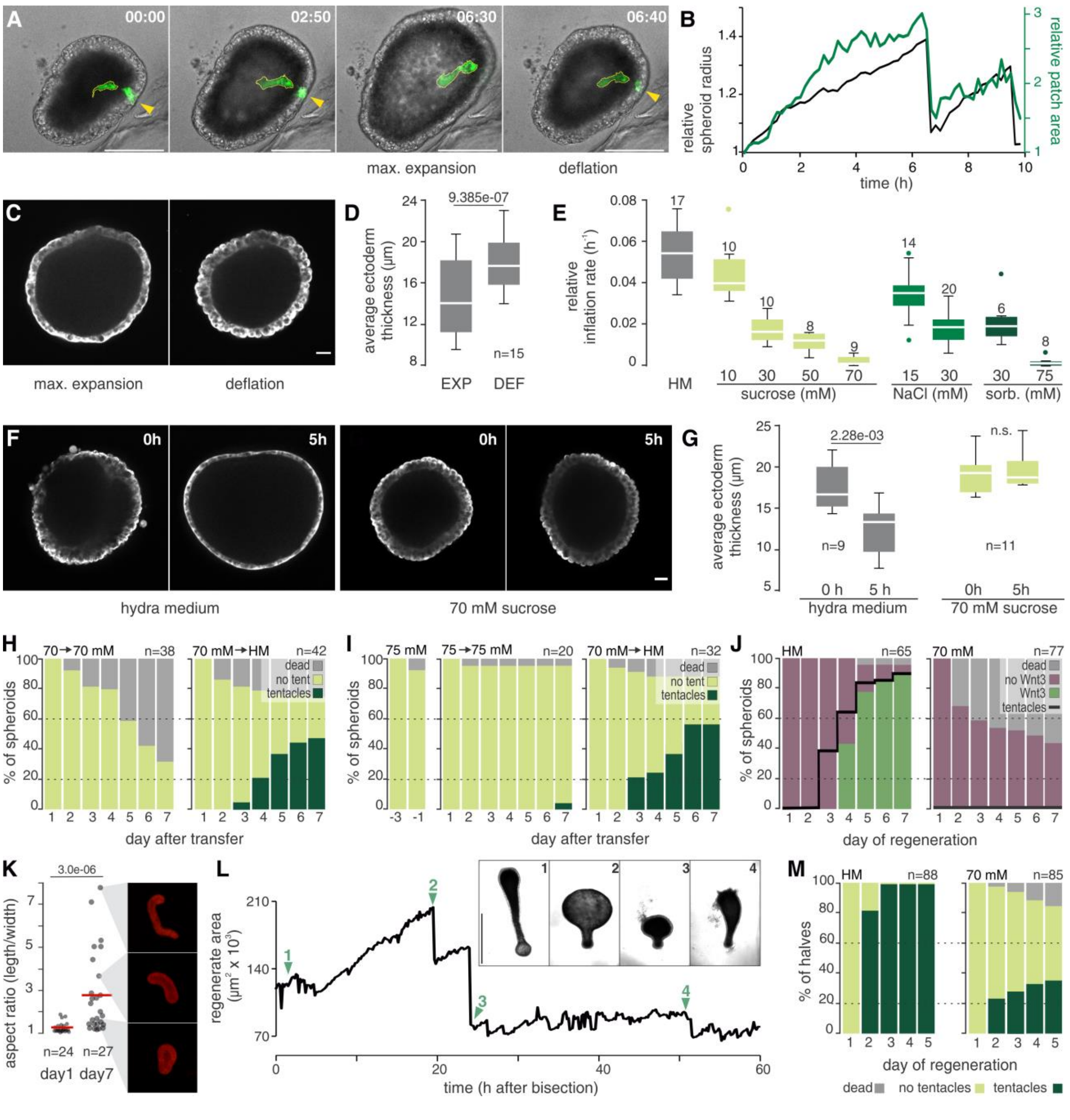

Fig. S1. Mechanical oscillations are required for *Hydra* regeneration

(**A-B**) Individual cells are being stretched during the spheroid expansion. (A) Snapshots of an example oscillating spheroid containing GFP-labelled cells (maximum Z projection of the fluorescent channel overlayed on brightfield images, scale bars 100 µm). Note the concomitant expansion of the green 2-cell patch with the spheroid inflation and its abrupt shrinkage after deflation. As the spheroid expands, another GFP+ cell (yellow arrowhead) moves out the the imaged volume. (B) Quantification of the spheroid radius and the cell area from (A). (**C-D**) Spheroid inflation causes tissue stretching, as evidenced by thinning of the epithelial layer in expanded spheroids. (C) An equatorial optical section of a representative spheroid, ubiquitously

expressing GFP in the ectoderm. Spheroid is shown fully expanded (left) and immediately after deflation (right). The time interval between the snapshots was 10 min; scale bar 20 µm. (D) Quantification of tissue thickness before (EXP) and after deflation (DEF) in a population of spheroids. (**E**) Spheroid inflation rate decreases as the osmolarity of the medium increases. Note that this effect manifests independently of the used osmolyte. The number of samples quantified in each condition is indicated above the boxes. See also Fig S2i for details of slope quantification. (**F-G**) Isotonic conditions prevent tissue stretching. (F) Equatorial optical sections of GFP(ecto) spheroids in different media immediately after closing (0h) and 5h later. Note that while the tissue is significantly thinner in the expanding spheroid in *Hydra* medium, no such effect is observed in isotonic medium (70 mM sucrose). Scale bar 20 µm. (G) Quantification of the ectoderm thickness in spheroids from experimental setup shown in (F). (**H**) Regeneration resumes in HM after previous incubation in isotonic medium (70 mM sucrose) for 72 h. (**I**) Experimental setup identical to (H), but using 75 mM sorbitol to create isotonic conditions. (**J**) Quantification of morphological regeneration (tentacle appearance) and molecular symmetry breaking (Wnt3 spot) in a *Wnt3::GFP* reporter line spheroids under control (HM) and isotonic conditions (70 mM sucrose). (**K**) The aspect ratio (length/width) of spheroids in isotonic conditions at the beginning and end of the regeneration time course. N=3 independent experiments. (**L**) Example of an oscillatory behavior in a head-regenerating half of a biseceted animal (Quantification of Movie S3) representative of n=11 samples. Numbered insets correspond to the numbered time points indicated by arrowheads in the plot. To allow clear imaging of the oscillations, without the interference of active regenerate movement, *Hydra* medium with 1 µM linalool was used. (**M**) Head regeneration in the headless halves of bisected animals in control vs. isotonic conditions. Animals were bisected at 50 % body length. Panels (H-J) and (M) show cumulative plots of n samples from 3 independent experiments. P-values in (D) and (G) were calculated using a one-sided paired Student's t-test, in (K) using the Wilcoxon rank-sum test.

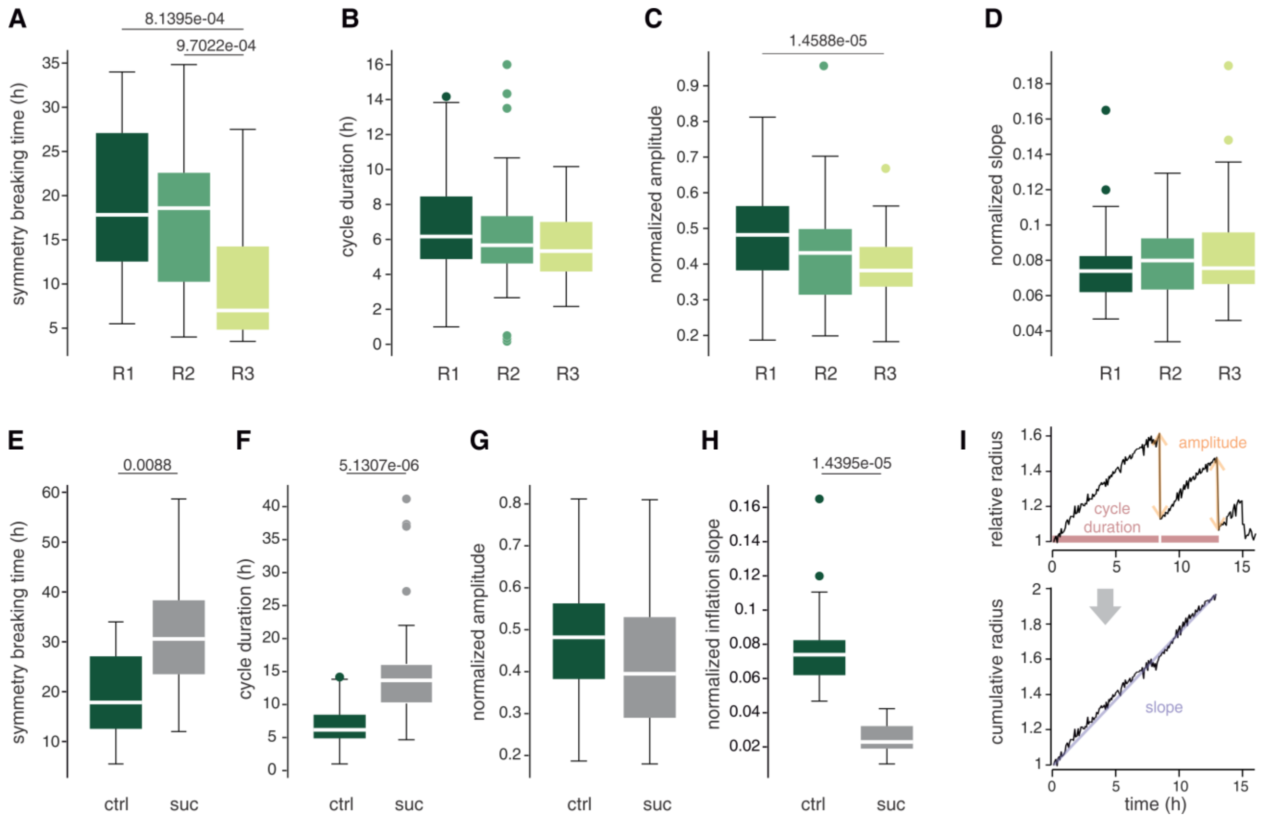

Fig. S2. Quantification of mechanical oscillation parameters

(**A**) Symmetry breaking time, measured as the time of Phase I to Phase II transition for spheroids derived from different axial positions (R1-R3) in control conditions. (**B**) Period of oscillations for the same samples. (**C**) Amplitude of oscillations for the same R1, R2 and R3 samples, indicating the maximum size of the spheroid in each cycle before it ruptures. Note the slight axial gradient of amplitudes, which might suggest a gradient in the resistance of the tissue to rupture. However, this gradient alone is not enough to explain the much more pronounced differences in the requirements for mechanical stimulation among the pieces. (**D**) Slope of inflation. Data shown here (A-D), correspond to the samples in Fig. 2A and are measured on spheroid radius data normalized to the initial size. (**E**) Comparisons of symmetry breaking time for R1 spheroids in control (HM, for samples in Fig. S2A) and in 30 mM sucrose. Similar comparisons for oscillation duration (**F**), oscillation amplitude (**G**) and inflation slope (**H**) which, as expected, decreases significantly, resulting in an increase of the oscillation period. (**I**) An example of the measured parameters. Note that the period and amplitude are quantified for each individual oscillation cycle, while the slope is measured for the entire Phase I, ignoring the deflation events. See (*48*) for details of the quantification procedure. Statistical comparisons were done using the Mood's median test. When significant, P-values are given directly in the plots.

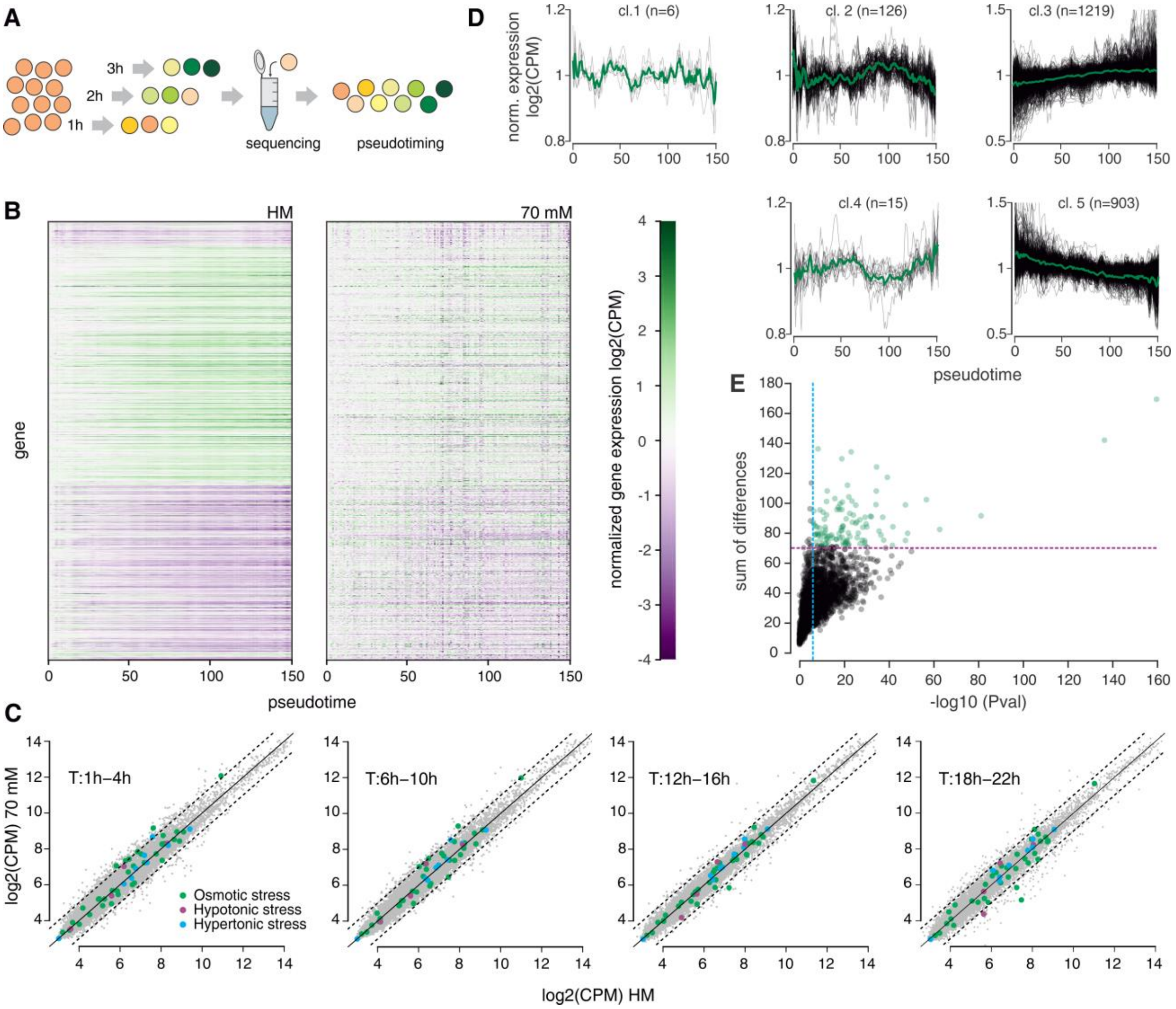

Fig. S3. Time series gene expression profiling for spheroids in control (HM) and isotonic (70mM sucrose) conditions

(**A**) Schematic of the experimental design. Spheroids were left to develop either in HM or isotonic medium after cutting. 8 spheroids were sampled every hour, collected and submitted individually to RNA-sequencing. Transcriptomics data later allowed to reconstruct the temporal development of spheroids (pseudotime) based on the similarity between samples. (**B**) Heatmaps of the temporal progressions for 2269 genes that change their expression at least 2-fold during the course of normal regeneration. Genes are clustered based on their behavior in control conditions. Note that in the isotonic conditions (70 mM sucrose) some of these genes behave differently (presented in Fig 3D), while others remain unaffected. In both datasets, the initial $\log_2$(CPM) values were subtracted for each gene. (**C**) Scatter plots of transcript counts in the isotonic conditions plotted against the control. Data for the indicated time points were averaged. Each dot corresponds to one gene. Genes involved in osmotic stress are highlighted in different colors based on their GO process annotation. Dotted lines indicate 2-fold up- or downregulation. The identity of the highlighted genes is given in Table S1. (**D**) Plots of the average behavior of the 5 gene clusters identified with hierarchical clustering in the control (HM) sample time-series. Black lines show individual genes, while the

thick green line is the cluster average. Numbers in parentheses indicate the number of genes in each cluster. (**E**) Selecting the top 10 % mechanosensitive genes. Only genes with a p-value < 10^{-6} for temporal changes in the difference between conditions were considered significant (blue line). Purple line indicates the magnitude-based cutoff (sum of differences between the conditions) for the top 10% of significantly changing genes (green dots).

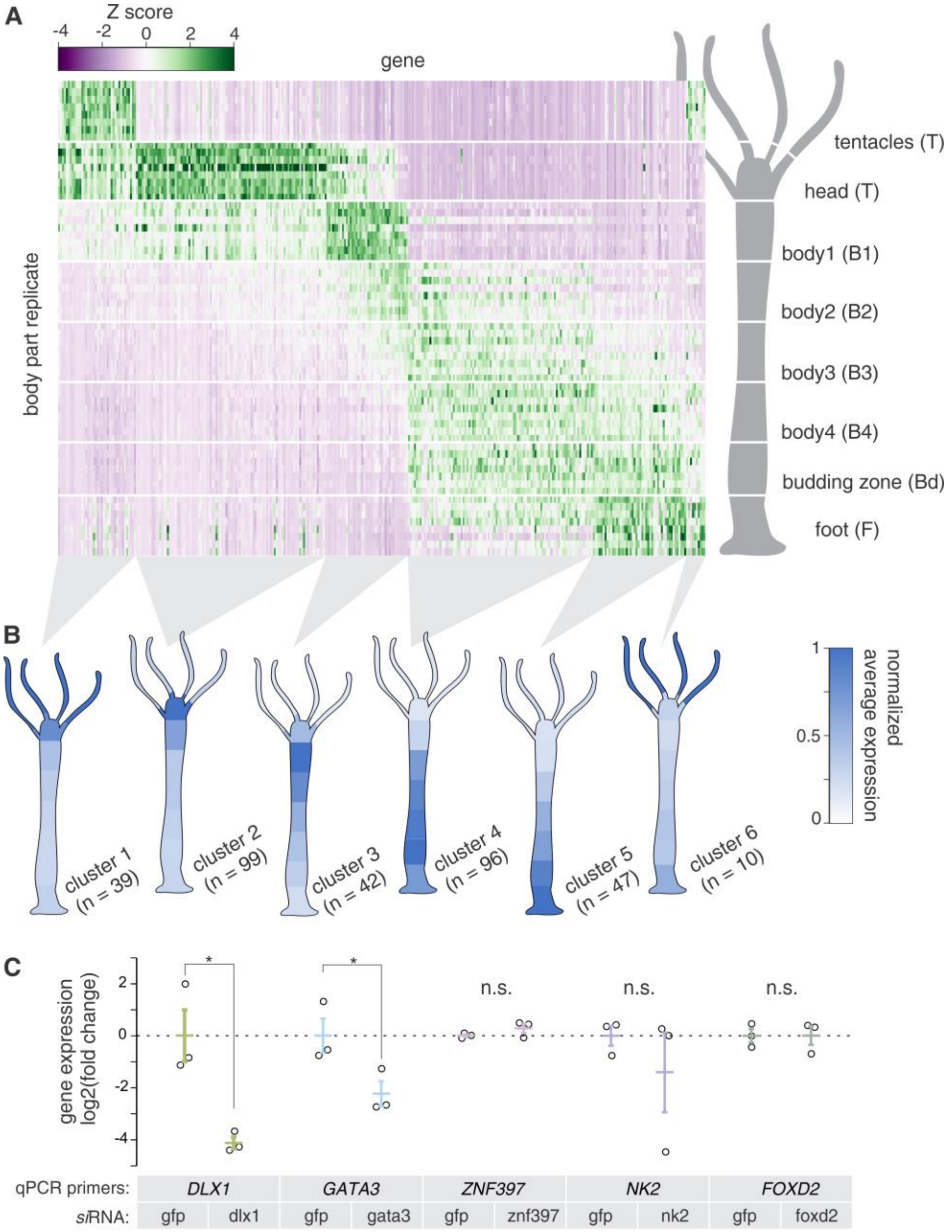

Fig. S4. Gene expression along the *Hydra* Oral/Aboral axis

(**A**) Heatmap of genes with axially graded expression, identified in the positional RNA-sequencing. Rows correspond to individual replicates for each body position. Color coding indicates the Z-score (standard deviations above or below the mean expression of a gene across all segments after collapsing biological replicates). These data were used to generate the PCA map of different positional identities in Fig3A-C. (**B**) Clusters of differentially expressed genes along the O/A axis, identified with k-means clustering. The schematics below show average expression patterns for each cluster, normalized to the body part with highest expression levels. (**C**) Q-PCR quantification of the expression of foot-specific transcription factors upon RNA-interference. Three replicates, each containing 3 regenerated halves per sample, were performed. Animals were bisected at 50% 2 days after the last siRNA electroporation and harvested for RNA extraction 3 days post bisection. Asterisk indicates a P-value < 0.05 from the Wilcoxon rank-sum test

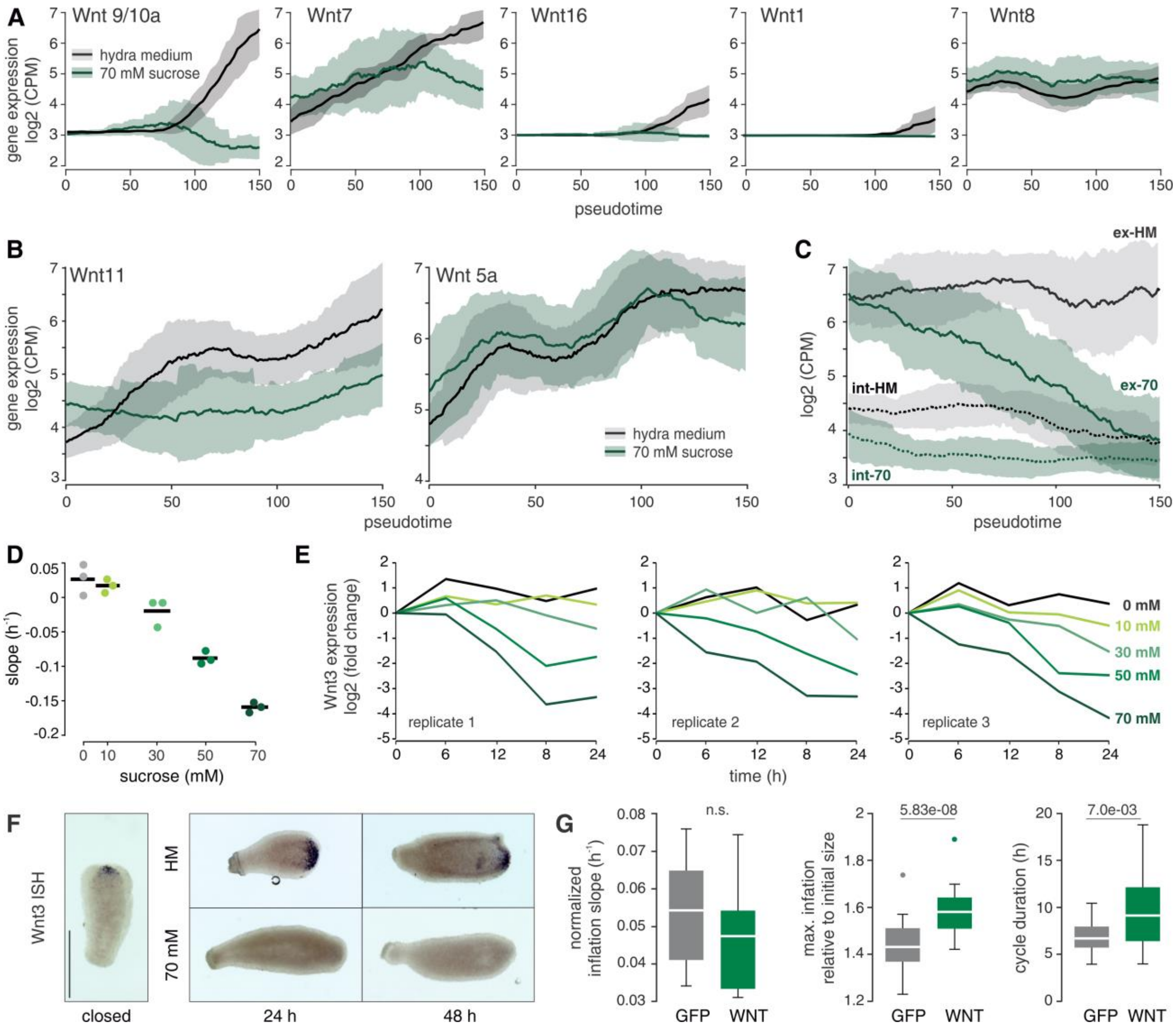

Fig. S5. Wnt signaling depends on mechanical stimulation

(**A**) Temporal gene expression profiles for genes shown in Fig. 6A. **(B**) Differential requirements of mechanical stimulation for the expression of non-canonical Wnt signaling ligands. While the expression pattern of Wnt11 (Wnt/PCP pathway) is sensitive to the removal of mechanical oscillations, Wnt5a (Wnt/Ca^{2+} pathway) does not show changes in the pattern of expression. (**C**) Exonic (solid lines) and intronic (dotted lines) counts for the WNT3 trascript in control (black) and isotonic (green) conditions. Lines in A – C show a moving mean over the temporal (pseudotime) gene expression data and the shaded area represents moving standard deviation. The size of the moving window is 36 pseudotime points. (**D**) Slopes of straight lines fitted on the Wnt3 expression data from Fig 5D. Dots indicate measurements in individual replicates, and lines represent averages. (**E**) Plots of individual replicates of the q-PCR timecourse of WNT3 expression in different osmolarities. For each plot, the lines indicate averages of 3 technical replicates. (**F**) Whole-mount in situ hybridization for the Wnt3 transcript in head-regenerating halves of bisected animals under control and isotonic (70 mM sucrose) conditions. Bisected halves were allowed to close the wound for ~ 1.5 h (closed) and then randomly split between the different

media. Representative data are shown. (**G**) Quantification of oscillation parameters in the *ecto pAct::GFP, pAct:Wnt3* spheroids (WNT, n=15). Spheroids from the line *ecto pAct::GFP* were used as a control (GFP, n=17). Wilcoxon rank-sum test was used for statistical comparison.

Table S1. Genes with the osmotic stress functional signature examined in Fig. S3C.
Shading in the table corresponds to the figure colors. The mammalian functional annotation data were acquired form the Molecular Signatures Database (https://www.gsea-msigdb.org/gsea/msigdb/). Corresponding *Hydra* genes were identified using the homolog annotation taken from the *Hydra*2 genome project (https://research.nhgri.nih.gov/*Hydra*/) and the NCBI genome database (https://www.ncbi.nlm.nih.gov/gene). Note that in some cases several *Hydra* genes map to an identical mammalian homolog.

NCBI GeneID	Mammalian homolog	Functional annotation
100197804	*AKR1B1*	Hypertonic response
100214195	*HNMT*	Hypertonic response
100199336	*LETM1*	Hypertonic response
100210191	*MICU1*	Hypertonic response
100198865	*OXT*	Hypertonic response
100198028	*SLC12A2*	Hypertonic response
100208923	*XRCC5*	Hypertonic response
100211211	*XRCC6*	Hypertonic response
105848752	*YBX3*	Hypertonic response
100209595	*CAB39*	Hypotonic response
100198551	*MYLK*	Hypotonic response
100197110	*OXSR1*	Hypotonic response
100210915	*SLC12A6*	Hypotonic response
100202442	*ABCB1*	Response to osmotic stress
101241118	*ABCB1*	Response to osmotic stress
100212701	*ANXA7*	Response to osmotic stress
101238969	*ANXA7*	Response to osmotic stress
105844932	*AQP9*	Response to osmotic stress
100203331	*AQP9*	Response to osmotic stress
100211688	*ATF2*	Response to osmotic stress
100211866	*ATF2*	Response to osmotic stress
100202650	*BAX*	Response to osmotic stress
100203439	*BAX*	Response to osmotic stress
101235784	*CAPN3*	Response to osmotic stress
100204696	*CASP3*	Response to osmotic stress
100212685	*CASP3*	Response to osmotic stress
100202915	*CASP3*	Response to osmotic stress
100198658	*DDX3X*	Response to osmotic stress
100192294	*DDX3X*	Response to osmotic stress
100200171	*DDX3X*	Response to osmotic stress
100213362	*DYSF*	Response to osmotic stress
100208455	*KCNMA1*	Response to osmotic stress
100204742	*KMO*	Response to osmotic stress
100201328	*MAP2K7*	Response to osmotic stress
100213972	*MDR1*	Response to osmotic stress

100199733	*MYLK*	Response to osmotic stress
100211813	*NOLC1*	Response to osmotic stress
101235318	*P53*	Response to osmotic stress
100197078	*PK1L2*	Response to osmotic stress
100207801	*PKD2*	Response to osmotic stress
100198291	*PKD2*	Response to osmotic stress
100207545	*PKD2*	Response to osmotic stress
100206298	*S12A2*	Response to osmotic stress
100204936	*SERPINB6*	Response to osmotic stress
100215837	*SORD*	Response to osmotic stress
100203619	*TP53*	Response to osmotic stress
100215316	*WNK3*	Response to osmotic stress
100205438	*ZFP36L1*	Response to osmotic stress

Table S2. Top 10% genes sensitive to the removal of mechanical stimulation. The annotation of mammalian homologs is taken from the *Hydra*2 genome project and the ncbi genome database. Only genes, for which a mammalian homolog could be found, were used in downstream GO terms analysis. Cluster identities correspond to those in Fig S3b-c.

NCBI GeneID	Mammalian homolog	Adjusted P value	Sum of differences (A.U.)	Cluster
100198792	*NPC2*	4.6326E-09	136.092381	5
100205069		6.9999E-24	134.095417	5
100213913		3.1262E-35	123.973407	5
100204588	*DMRT1*	3.6722E-13	107.95492	5
100200513	*H4-16*	1.6495E-57	102.175059	5
100200175	*PAX3*	3.2117E-24	101.235388	5
100197450		7.4321E-13	100.906573	5
100210977		7.4045E-10	95.3031152	5
100211420		3.3403E-23	92.9587732	5
101235091	*BPI*	7.3336E-25	91.9228974	5
105847657		4.6034E-30	90.4197589	5
100200125		6.7357E-12	89.0720116	5
100204140	*SLC36A1*	3.6431E-20	84.6764672	5
105846366		3.1009E-12	83.8653636	5
101235129		7.1214E-10	83.1229474	5
105846591		2.1964E-12	81.9765514	5
100204267	*H4-16*	2.5964E-36	81.1650399	5
100192253	*MDN1*	7.8772E-10	79.260342	5
101237889		6.3432E-09	79.0574564	5
100215610		1.1107E-07	78.8610029	5
101241797		9.9278E-08	78.6866458	5
100214145	*ARF1*	3.3494E-37	78.4883676	5
100210142		2.5949E-09	78.1921106	5
100204912	*FAXC*	3.6078E-13	77.8118357	5
105848556		7.8388E-19	76.6290038	5
100205153		4.0525E-13	76.6027665	5
100198375	*UBE2M*	3.0794E-10	75.8575807	5
101239409	*CHST11*	6.3026E-13	75.6299749	5
105845131		4.9111E-22	75.131633	5
105850420		6.0134E-30	74.830474	5
101240945		3.0763E-21	73.637984	5
100205795	*HEXA*	2.8568E-12	73.0806362	5
100207977	*CHAC1*	7.7933E-11	72.7090957	5
100206276	*SPIRE1*	7.7358E-28	72.6416818	5
100204652	*M1AP*	1.5981E-10	71.0456557	5
100213095	*CHST1*	3.3537E-15	70.7990643	5
100205814		6.5178E-20	100.53371	4

105850674		6.6341E-07	87.1054945	4
100210377		5.7241E-12	80.1938687	4
105847121		8.6105E-20	77.1119522	4
100215038	*WNT9/10a*	1.809E-160	169.212235	3
100214793		3.717E-137	141.847658	3
101234798	*MMP19*	1.1136E-19	129.449635	3
100211747	*HPGDS*	4.6766E-40	117.071199	3
100214868	*WNT7*	3.2928E-22	111.795984	3
100212279		4.1605E-35	107.875029	3
100203050	*WNT3*	7.9199E-23	104.022471	3
100206447	*DDR1*	1.3286E-16	102.877443	3
100209110		5.9247E-19	99.3826011	3
101240588	*COL4A1*	5.6316E-35	98.8317157	3
105847477	*KLKB1*	3.9643E-48	98.6953477	3
105843060	*MTPN*	5.8948E-26	98.4730393	3
105846947	*DMBT1*	4.3646E-18	97.1788779	3
100201149	*GOLGB1*	1.1656E-18	95.571484	3
100199145		9.7521E-20	95.5645877	3
100213338		1.6442E-17	95.4806636	3
100203315		3.821E-07	94.5783671	3
100215832		2.6035E-11	93.8196351	3
100210883	*ZIC4*	7.0249E-13	92.0023137	3
100198091		5.6989E-82	91.3725492	3
100205589	*ZSWIM6*	1.9592E-32	91.0877752	3
101235638	*MC1R*	5.9506E-33	90.7801104	3
100215883		5.4484E-38	90.4575232	3
100214647	*COL4A2*	1.2705E-21	89.3511821	3
100197980	*AGO2*	8.6049E-28	88.7448567	3
100197276	*LAMB1*	2.3072E-26	87.2690407	3
100215810	*MUC5AC*	2.1317E-24	87.0480783	3
100208937		6.3469E-26	86.3673867	3
100211987	*COL1A1*	1.4675E-30	85.9500703	3
100213961		3.2435E-07	85.6512258	3
105843397	*SUCNR1*	5.8369E-08	84.5183252	3
100200109	*AGO1*	3.3473E-20	84.2078428	3
100205317	*BCR*	1.5419E-42	84.0707399	3
100201345		6.9752E-31	83.9326117	3
105843191	*SMYD4*	1.4373E-08	83.900393	3
100209165	*PXN*	4.0241E-29	83.720318	3
101237470	*SP5*	1.6895E-19	83.4839872	3
100203539	*SBF2*	1.8026E-63	82.2574124	3
100212732	*ZNFX1*	6.561E-20	81.8082977	3
100200618	*LAMA3*	2.2243E-19	81.5123445	3

100205218	*COL4A1*	1.2508E-22	80.0568453	3
100211594	*SH3RF1*	4.5916E-27	79.8703778	3
100214250	*COMP*	4.0807E-49	79.7026634	3
100205822		8.7407E-11	79.6388417	3
100214706	*ZNFX1*	1.3698E-08	79.6088021	3
105847351		1.9103E-18	79.5634631	3
105847324		3.6577E-15	78.590009	3
100203271		2.2374E-14	77.9479218	3
100204823		5.0267E-12	77.8807991	3
101234364	*ZNFX1*	2.9224E-23	77.5378183	3
100204347		1.5128E-26	76.8581599	3
100211037	*PITX1*	1.9622E-15	76.6511158	3
100202525		3.0872E-17	75.9005866	3
100207920	*TIMP3*	3.1925E-17	75.6159102	3
101240985	*COL6A6*	8.2395E-29	75.1719549	3
100208925	*TRPV3*	1.9281E-38	75.1135063	3
101239940	*HECTD1*	3.9643E-48	74.9103455	3
101240505	*COL4A1*	1.4454E-21	74.5043193	3
100215811	*ANKRD52*	1.3513E-13	74.1728462	3
105844127		4.9043E-22	73.8252026	3
105849579		3.1318E-08	73.7209731	3
100215286	*COL6A6*	3.3727E-20	73.6005058	3
100205070		6.2009E-30	73.3631814	3
100200555		1.0528E-12	72.4081638	3
100207691	*LAMC1*	8.1635E-23	72.3880374	3
100199754	*COL1A1*	3.3394E-21	72.28531	3
101239232	*ANKRD28*	1.9786E-20	71.8506799	3
100209738	*SF3B1*	2.3597E-42	71.6429143	3
100209752		1.3275E-10	71.6130722	3
100206389	*AXIN1*	1.545E-22	71.4835447	3
100202503		9.9044E-09	71.324878	3
101239976	*TFPI*	1.9211E-17	70.8434945	3
100205900	*BANK1*	5.3075E-30	71.5096716	2

Table S3. Functional annotation enrichment of the upregulated and downregulated genes from the top 10 % mechanosensitive genes. Only the dominant clusters of genes were analyzed since the number of members of the other clusters within the set was negligible and not sufficient to analyze enrichment. Green shading indicates functional terms related to extracellular matrix and yellow shading indicated functional terms related to Wnt signaling. Note that there were no retinoic acid signaling genes. The enrichment of this signature is generated by Wnt signaling genes, as they often crosstalk to the retinoic signaling. No significantly enriched functional signatures were found for cluster 5.

upregulated in control (cluster 3)	
Functional category	False discovery rate
extracellular matrix part	3.80E-13
extracellular matrix structural constituent	1.66E-10
endoplasmic reticulum lumen	1.48E-09
frizzled binding	6.43E-09
collagen catabolic process	5.38E-08
multicellular organismal catabolic process	1.01E-07
collagen metabolic process	4.34E-07
multicellular organismal macromolecule metabolic process	5.77E-07
multicellular organismal metabolic process	8.98E-07
basement membrane	9.62E-07
cell fate commitment	2.32067E-06
gene silencing by RNA	3.9343E-06
skeletal system development	1.01115E-05
Wnt signaling pathway	1.30022E-05
response to retinoic acid	1.42522E-05
collagen	2.1533E-05
platelet-derived growth factor binding	2.46334E-05
gene silencing by miRNA	3.37478E-05
basal lamina	3.37921E-05
posttranscriptional gene silencing by RNA	4.85417E-05
posttranscriptional gene silencing	4.85417E-05
gene silencing	4.85456E-05
receptor agonist activity	5.83005E-05
cellular response to retinoic acid	5.86821E-05
G-protein coupled receptor binding	0.000107706
Golgi lumen	0.000151083
forebrain development	0.000158994
canonical Wnt signaling pathway	0.000195776
downregulated in control (cluster 5)	
Functional category	False discovery rate
intracellular lipid transport	0.124710452
amino acid transport	0.188127412
sulfur compound metabolic process	0.242697813

organic anion transport	0.477999928
carnitine transmembrane transport	0.498036189
organic acid transport	0.498036189
carboxylic acid transport	0.498036189
amino-acid betaine transport	0.498036189
carnitine transport	0.498036189
biotin metabolic process	0.498036189

Movie S1.
Control spheroid imaged in *Hydra* medium.

Movie S2.
Control spheroid imaged in isotonic medium with 70 mM sucrose.

Movie S3.
Oscillations of a head-regenerating half from a bisected animal in *Hydra* medium.

Movie S4.
Wnt-overexpressing spheroid imaged in *Hydra* medium.

Movie S5.
Wnt-overexpressing spheroid imaged in isotonic medium with 70 mM sucrose

Movie S6.
Bud spheroid imaged in *Hydra* medium.

Movie S7.
Bud spheroid imaged in isotonic medium with 70 mM sucrose
